# Advances in the Direct Nanoscale Integration of Molecularly Imprinted Polymers (MIPs) with Transducers for the Development of High-Performance Nanosensors

**DOI:** 10.3390/bios15080509

**Published:** 2025-08-06

**Authors:** Ibrar Muhammad Asif, Tiziano Di Giulio, Francesco Gagliani, Cosimino Malitesta, Elisabetta Mazzotta

**Affiliations:** Dipartimento di Scienze e Tecnologie Biologiche ed Ambientali (Di.S.Te.B.A.), Università del Salento, Via per Monteroni, 73100 Lecce, LE, Italy; muhammadibrar.asif@unisalento.it (I.M.A.); tiziano.digiulio@unisalento.it (T.D.G.); francesco.gagliani@unisalento.it (F.G.); cosimino.malitesta@unisalento.it (C.M.)

**Keywords:** molecularly imprinted polymers (MIPs), nanosensors, nanotransducers

## Abstract

Molecularly imprinted polymers (MIPs) have emerged as robust, cost-effective analogues of bioreceptors, offering high selectivity and stability. When applied in sensors, one key step is the integration of MIPs with the transducer, which critically affects sensor performance. Demanding challenges come when such integration involves nanoscaling processes, meaning that the transducer is nanostructured or the MIP itself is nanosized on a bulk transducer. In both cases, the integration results in the development of nanosensors, with advantages arising from the nanoscale, such as a high MIP surface-to-volume ratio, with surface-located, easily accessible binding sites, fast binding kinetics, and, thus, a rapid sensor response. Major advantages come also from nanostructured transducers, with nanoscale geometry enabling highly sensitive signal generation processes, not allowed on their bulk counterparts. In this review, we discuss advances in imprinting technologies, focusing on techniques that, enabling the nanoscale control of MIP synthesis, are conveniently applied to directly integrate MIPs with nanosensors in a one-step process. Two main approaches are reviewed, consisting in MIP nanostructuring on bulk transducers and in the direct growth of MIPs on nanotransducers, highlighting how different strategies achieve good conformity at the nanoscale and address spatial complexity to ensure stable and accurate signal acquisition. Finally, we consider future directions in MIP-based nanosensor development.

## 1. Introduction

**Figure 1:**
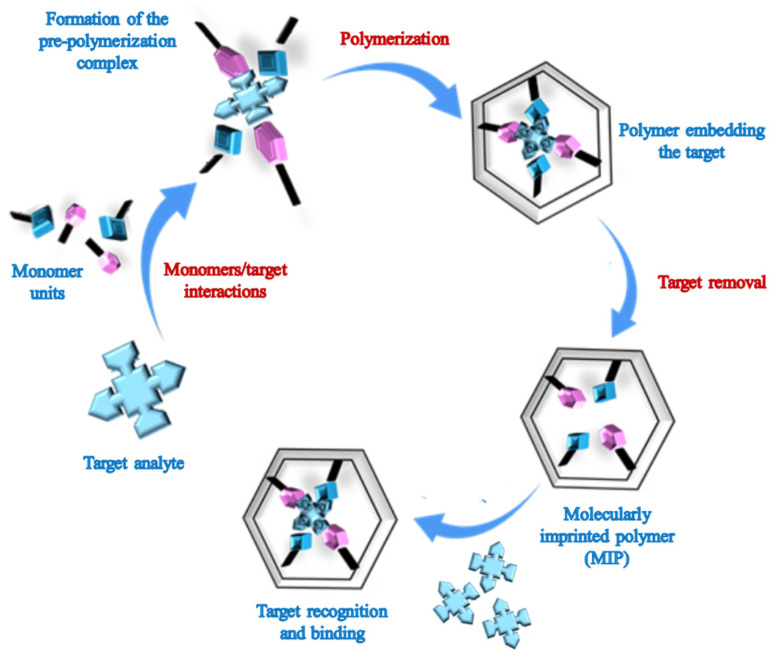
Molecular imprinting procedure. From the pre-polymerization complex to the MIP receptor.

Molecularly imprinted polymers (MIPs) are synthetic polymeric receptors produced to have high affinity for a specific target molecule [1,2,3,4,5,6,7,8]. At the heart of MIP technology is a molecular imprinting process: a polymer network forms around a target molecule (the template), and once the template is removed, complementary binding cavities remain within the polymer matrix, which ensure high affinity and selectivity toward the original target (Figure 1) [6,7,8]. Each cavity features the precise spatial arrangement of complementary functional groups, enabling the MIP to “remember” the target’s stereochemistry and binding mode [9,10]. The main components in MIP synthesis are the polymerizable functional monomers and the template molecule, between which several interactions can occur, such as strong covalent bonds—less used today—or weaker non-covalent interactions, which determine the choice of the proper protocol for template removal [11]. The polymerization mixture may also contain a crosslinking agent, whose role is to fix the polymer structure around the target template, and a porogenic solvent, ensuring the solubilization of all the components and determining the resulting polymer porosity.

The large success achieved by MIPs during the last decades is due to their ability to combine the selectivity of biological receptors, simulating the “lock-and-key” mechanism, with the stability and low cost of synthetic polymers [12,13]. Early work by Wulff (1972) and Mosbach (1980s) established this concept [14,15,16]. As Mosbach noted, the polymers obtained display a high degree of stereo- and regiochemical selectivity, making applications in chiral separations and as “antibody mimics” (so-called plastic antibodies) feasible. Indeed, MIPs are often described as robust analogues of natural antibodies [17,18].

Due to their properties, MIPs have found applications across many fields, including separations, solid-phase extraction, and chromatography [19,20,21,22,23]. They are also used in drug delivery and release systems (to encapsulate and release pharmaceuticals in a targeted way). Importantly for this review, MIPs serve as the recognition element in numerous chemical sensors [18,24,25,26], where they are paired with a reporting system, which may be electrical, electrochemical, optical, or gravimetric, and the presence of the target affects the reporter output.

A crucial challenge in MIP-based sensor design is integrating the imprinted polymer with the chosen transducer [27]. In general, two approaches can be used, consisting of the immobilization of pre-formed MIPs (often as polymer particles or nanoparticles) or of the in situ synthesis of the MIP directly on the transducer surface. The first approach allows for the independent optimization of MIP polymerization (monomer ratios, porogen, etc.) and the sensor assembly steps, thus providing, in principle, higher flexibility to both. For example, MIP particles can be physically adsorbed, embedded in a membrane, or chemically coupled via surface linkers to an electrode or fiber [28,29,30,31,32], with the possibility of incorporating additives (e.g., conductive carbon, metal nanoparticles) into the immobilization layer to boost sensitivity. On the other hand, this approach requires suitable surface chemistries on both the MIP and transducer surface, which often means introducing specific functional groups (–NH_2_, –COOH, –SH, etc.) to enable conjugation processes [26,27]. These procedures not only make the sensor assembly more complex and time-consuming, but they may not be suitable when nanostructured transducers (e.g., nanostructured electrodes, photonic architectures, nanoporous materials, plasmonic nanostructures) are used, with a possible mismatch between the nanoscale surface and the MIP size, thus impairing the target analyte binding.

In the case of an MIP’s direct integration with the transducer, the pre-polymer mixture (monomers, crosslinker, initiator, template) is directly applied to the transducer and polymerized in situ [33,34]. The resulting MIP film is grown in intimate contact with the transducer, yielding excellent adhesion and precise control over film thickness. In this way, a “ready-to-use” sensor is obtained in a single step: once the polymerization and template washing are complete, the device is immediately functional without further assembly.

Further performance gains arise when such direct MIP–transducer integration involves the nanosizing of the MIP and/or the transducer [35,36]. Nanosized MIPs have indeed an exceptionally high surface-to-volume ratio [36]. This exposes a large number of binding sites per unit mass, which greatly facilitates target capture, with enhanced sensor sensitivity, faster binding kinetics, and, thus, a reduced response time.

On the other hand, using nanostructured transducers can also boost sensing performance [37,38,39,40] due to their inherently high sensitivity arising from nanoscale-related processes. For example, the combination of MIPs with a photonic nanostructure has shown great interest [38] due their simple optical readout. These structures are formed by the periodic arrangements of materials possessing divergent dielectric constants, thus generating a photonic bandgap (PBG) that reflects light at specific wavelengths. Upon analyte attachment on the surface, a change in the periodic structure is induced, producing a change in the PBG and then the refractive index, allowing the binding event to be monitored by signal as a Bragg diffraction [41,42] or a Fabry–Perot pattern [43,44,45], which is not achievable on bulk transducers. Beyond photonic structures, nanoporous materials, such as gold, nickel, polymeric nanofilaments, and alumina, have been used as electrochemical transducing systems, proving their superior conductivity compared to conventional flat electrodes [46,47,48]; this conductivity originates from their larger surface area, which intensifies electron transfer. Also, TiO_2_ tubular nanostructures have been used for MIP-based nanosensor development [49,50,51] due to their hollow structure, promoting analyte transport, and their excellent photoelectrochemical behavior, ascribed to nanotitania’s light-absorbing properties [52,53].

In MIP-based nanosensors, stable and accurate signal acquisition relies on a reliable MIP–transducer integration at the nanoscale. For this purpose, achieving a highly conformal integration preserving the nanofeatures of the transducer is required, leading to a MIP which perfectly replicates the nanostructured transducer surface. This can therefore be achieved by two main strategies, one involving the nanosizing of MIPs on bulk transducers and the other exploiting direct MIP deposition techniques on nanostructured transducers.

Recently, several valuable reviews have been published on the development of MIP-based nanosensors [54,55,56,57], including those focused on wearable and flexible devices [56]. While these works offer comprehensive overviews—particularly regarding MIP deposition techniques onto generic transducers [54] and nanoparticle-based systems [57]—they tend to pay limited attention to the synthesis of nanosized MIPs and their direct integration with nanotransducers. In this context, our review aims to highlight the most recent strategies for synthesizing nanosized MIPs and coupling them directly with nanosized transducing systems, offering an updated and focused overview of the current state of the art. Specifically, MIP nanomolding [58] and nanopatterning [36] techniques are illustrated. In nanomolding approaches, sacrificial templates such as nanoporous anodic alumina membranes or silica nanoparticles are used to obtain MIPs in the form of nanofilaments, nanofibers, or nanostructured photonic crystals [41,42]. In contrast, nanopatterning typically involves lithographic methods at the nanoscale, offering high spatial resolution but increasing fabrication complexity. Although relatively few one-step strategies have been reported to date for direct MIP integration with nanotransducers, here, we describe the application of electro-polymerization [59], dopamine self-polymerization [60], localized photo-polymerization [61,62], and vapor-phase polymerization [43]. An overview of the most significant examples of both approaches is provided here, highlighting the benefits and challenges of each strategy, with the aim of emphasizing the enormous opportunities coming from the combination of imprinting and nanosensor technologies and to possibly stimulate new solutions and strategies able to respond to the growing demands and applications of chemical sensors.

## 2. Nanosized MIPs on Bulk Transducers

### 2.1. MIP Nanomolding

Nanomolding employs sacrificial, pre-patterned molds, such as anodic alumina membranes, colloidal silica arrays, or soft polymeric stamps, to define the nanoscale architecture of an MIP [63,64]. First, the pre-polymer mixture (functional monomer, cross-linker, initiator, porogen, and template molecule) is cast or infiltrated into the interstices of the mold. Upon polymerization, a rigid, cross-linked network forms within the mold nanostructure, whose subsequent removal (by chemical etching or solvent dissolution) unveils ordered arrays of polymer nanofilaments, nanodots, or inverse opal architectures complementary to the templating scaffold, surface-bound or free-standing in solution. In most cases, the template molecule is preliminarily immobilized on the scaffold surface so that the etching procedure also determines template removal at the same time and thus the formation of surface-located imprinted cavities.

**Figure 2:**
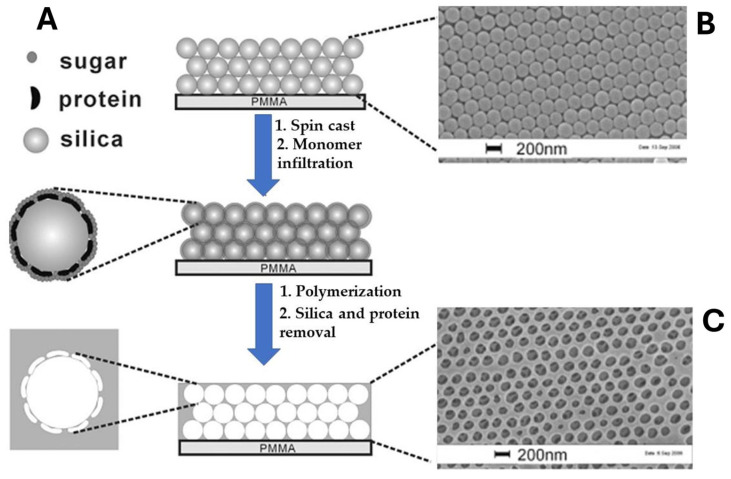
Schematic illustrating the fabrication of a protein-imprinted hydrogel film for the selective recognition of BSA. (**A**) 3D silica colloidal array deposited on PMMA by spin-coating; SEM images of (**B**) the silica-colloidal crystal template and (**C**) the resulting photonic MIP film with a 3D, ordered, interconnected macroporous structure. Adapted with permission from Ref. [41]. Copyright © 2007, Wiley-VCH GmbH.

This approach has been exploited extensively in optical MIP-based sensors [41,42]. In a seminal work by Hu et al. [41], photonic crystals were combined with surface molecular imprinting to construct a biosensor using BSA as template molecule (Figure 2). The template protein was anchored onto the surface of a 3D silica colloidal array by spin-casting, and a solution of saccharose was used to prevent the protein from denaturing. A solution containing methacrylic acid (MAA) as the functional monomer, ethylene glycol dimethylacrylate (EGDMA) as the cross-linker, and azobisisobutyronitrile (AIBN) as the initiator was infiltrated in the space of the silica array. After radical polymerization, the silica nanoparticles with the anchored protein were removed, yielding hydrogel films with an inverse opal structure exhibiting high sensitivity and selectivity toward BSA. Specifically, the binding of the target protein induced a reversible swelling of the hydrogel matrix in aqueous media, leading to measurable shifts of the Bragg diffraction peak and visible color changes, attributed to alterations in the effective refractive index. This platform achieved detection limits in the ng/mL range. To demonstrate the versatility of the approach, the same research group imprinted other molecules, such as theophylline and ephedrine, with the sensor, which was able to reach very low detection limits (pg/mL) and a fast response time of 20 s [42].

**Figure 3:**
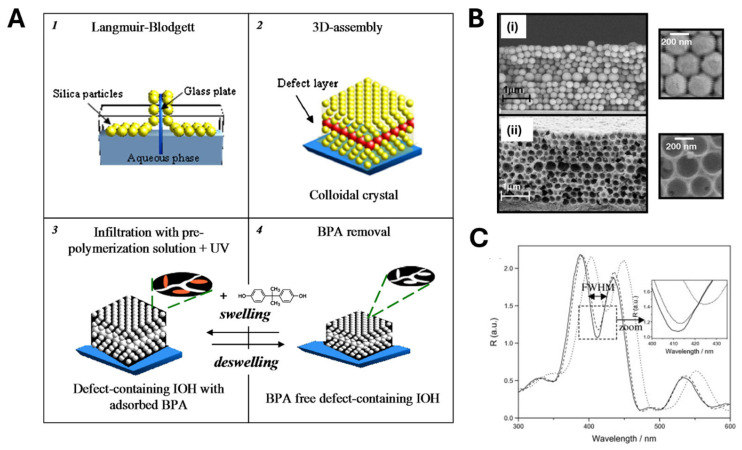
(**A**) Schematic illustration of the procedure used for the preparation of a molecularly imprinted inverse opal hydrogel film containing a 2D defect layer. (**B**) SEM images of (**i**) the colloidal crystal template with an embedded planar defect layer and (**ii**) the resulting BPA-imprinted photonic polymer film. (**C**) Sensor signal after BPA extraction (full line) and upon soaking in buffer solutions with BPA 10^−9^ M (dashed line) and 10^−3^ M (dotted line). The inset shows a zoomed-in view of the λ_max_ shift as a function of BPA concentration. Adapted with permission from Ref. [70]. Copyright © 2011, Elsevier.

Several research teams have drawn inspiration from Hu’s work to develop sensing platforms based on inverse opal structures [65,66,67,68]. For instance, Chen et al. fabricated a molecularly imprinted photonic hydrogel for selective L-histidine (L-His) detection [69]. Using a similar procedure, they created an inverse opal film whose Bragg diffraction peaks shifted significantly more in the presence of L-His than for competing amino acids. This sensor achieved a 10 pM detection limit and responded within 60 s. Furthermore, it could be regenerated by washing in acidic medium and was successfully applied to L-His determination in beverage samples. Griffete et al. [70] engineered a molecularly imprinted inverse opal hydrogel (IOH) film that, unlike the previous examples, contained a 2D “planar defect layer” that conferred optical properties to the sensing platform (Figure 3). First, a three-dimensional colloidal crystal template of silica microspheres (≈200 nm diameter) was assembled on a substrate via Langmuir–Blodgett deposition, creating an artificial opal with uniform pores. To introduce the planar defect, a layer of slightly larger and differently packed spheres was inserted at a defined depth, forming a “slice” of oversized pores that disrupted the crystal periodicity. Next, a UV-polymerizable mixture (monomers, cross-linker, initiator, and bisphenol A template) infiltrated all pores, and two-hour UV irradiation (365 nm) induced photo-initiated polymerization. Subsequent selective removal of silica spheres (via an etching procedure with HF) and template extraction yielded an inverse opal hydrogel (poly (MAA-co-EGDMA)) with uniform 200 nm cavities and a row of enlarged cavities at the defect plane (Figure 3B). When exposed to white light, this defect plane generated a sharp reflection peak—referred to as the defect mode—within the photonic bandgap of the surrounding periodic structure. Upon target rebinding, local swelling of the hydrogel near the defect region altered the material’s refractive index, resulting in a red shift of the defect-mode reflection wavelength and a concurrent narrowing of the full width at half maximum (FWHM), as illustrated in Figure 3 C [70]. Notably, the system exhibited full reversibility: removal of the target molecule induced hydrogel deswelling and a corresponding blue shift of the Bragg peak, restoring the initial optical properties.

Nanoporous substrates represent another scaffold commonly employed in MIP nanomolding. Ouyang et al. [71] fabricated polydopamine (PDA)-based MIP nanowires by first anchoring bovine serum albumin (BSA) onto a 200 nm pore anodic alumina (AAO) membrane and then polymerizing dopamine with ammonium persulfate in neutral phosphate buffer at 45 °C for six hours. They used dopamine as the monomer due to its ability to polymerize under mild conditions, yielding an adhesive polymer. After dissolving the alumina template in diluted HF, they obtained free-standing PDA–MIP nanowires that were used for BSA detection by UV–Vis spectroscopy, showing high selectivity.

**Figure 4:**
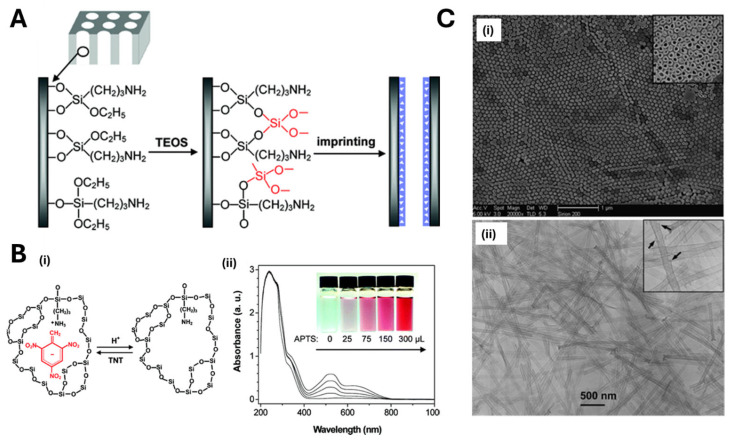
(**A**) Schematic of the TNT-imprinted silica nanotubes synthesis within alumina membrane pores. (**B**) (**i**) Schematic illustration for the molecular interaction of TNT with the MIP silica matrix through the anion−cation pair; (**ii**) color change produced changing the monomer concentration in the polymerization mixture. (**C**) SEM and TEM observations of TNT-imprinted silica nanotubes: (**i**) Top-view SEM image of the TNT-imprinted silica nanotube array embedded inside an alumina membrane after the membrane surface was mechanically polished; (**ii**) TEM image of individual TNT-imprinted silica nanotubes liberated from the alumina pores by dissolving the alumina membranes. Insets are high-magnification SEM and TEM images, respectively. Adapted with permission from Ref. [72]. Copyright © 2008, American Chemical Society.

Xie et al. [72] imprinted TNT into the walls of silica nanotubes obtained by sol–gel polymerization within alumina nanoporous membranes, used as sacrificial molds (Figure 4A). The alumina membranes were first modified with 3-aminopropyltriethoxysilane (APTS), then exposed to a polymerization mixture containing tetraethylorthosilicate (TEOS), the target TNT and APTS. The polymerization was performed in an oven at 60 °C for 24 h under nitrogen atmosphere, and then the material was cured in an oven at 150 °C for 6 h. After the dissolution of the scaffold, silica nanotubes were successfully obtained, as revealed by SEM and TEM images (Figure 4C). Target detection was achieved by exploiting the absorbance of MIP nanotubes in solution and its modification with a binding process, appreciable by the related color change (Figure 4B). The high temperatures used in the protocol could represent a drawback preventing its application to the imprinting of biomolecules.

**Figure 5:**
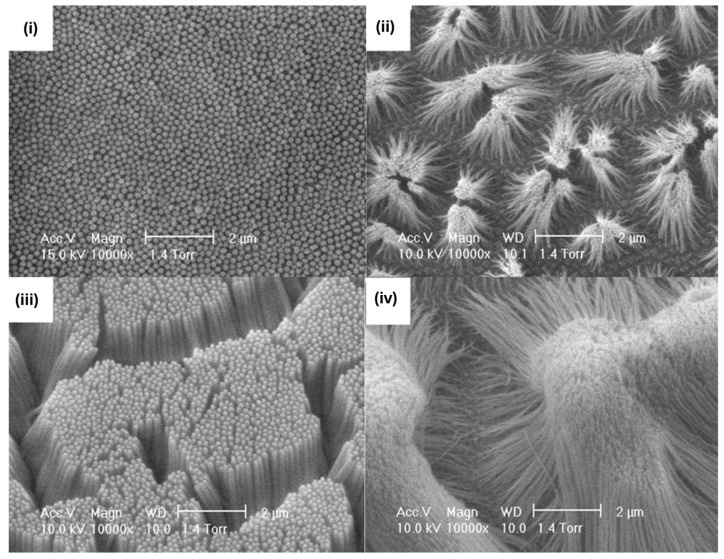
SEM images of the MIP nanofilaments obtained under different experimental conditions (electrooxidation/phosphoric acid treatment durations): 45 s/70 min (**i**), 1 min/15 min (**ii**), 4 min/70 min (**iii**), 4 min/15 min (**iv**). Adapted with permission from Ref. [73]. Copyright © 2007, Wiley-VCH GmbH.

Haupt’s group successfully combined photo-polymerization with nanomolding. In a seminal study [73], they generated MIP nanofilaments by a nanomolding approach, photo-polymerizing within the pores of a sacrificial porous alumina template. A layer of porous alumina—laid on a glass slide—was impregnated with a polymerization mixture containing trimethylolpropane trimethacrylate (TRIM), methacrylic acid (MAA), and the target molecule, propranolol. UV irradiation triggered co-polymer formation inside the alumina’s pore network; subsequent removal of the alumina template yielded arrays of surface-bound nanofilaments. By tuning the experimental conditions, namely the duration of the electrooxidation and of the treatment with phosphoric acid used to remove the alumina scaffold, they controlled filament diameters (50–200 nm) and lengths (0.5–5 µm) (Figure 5). The nanofilaments were then used to detect radiolabeled propranolol via liquid scintillation counting. In contrast to flat, featureless MIP films—which exhibited very low propranolol binding due to slow or hindered diffusion into buried sites—these high-aspect-ratio nanostructures showed dramatically improved recognition capabilities, since their high surface area facilitated rapid access to binding sites.

An extension of the method for the imprinting of small dyes and proteins was proposed by Linares et al. [58] who developed surface-bound MIP nanofilaments with high aspect ratios (≥40) using a similar nanomolding process on nanoporous alumina. In a subsequent work, Zdunek et al. [74] produced molecularly imprinted nanofilaments (MINs) for the luminescent detection of the antibiotic enrofloxacin (ENR). In this case, the analyte was first grafted onto the alumina surface, and polymerization was carried out under UV light (312 nm) for 15 min. After the alumina scaffold dissolution and the simultaneous removal of the target from the polymer matrix, the resulting MINs were able to selectively recognize ENR molecules. The binding event was monitored by taking advantage of the interaction between ENR and europium (III) ions, resulting in a highly luminescent ENR–lanthanide complex with strong narrow emission in the visible range. In this way, for target detection, MINs were first exposed to ENR and then to europium (III) ions, and the signal was monitored by UV-VIS spectroscopy [74].

**Figure 6:**
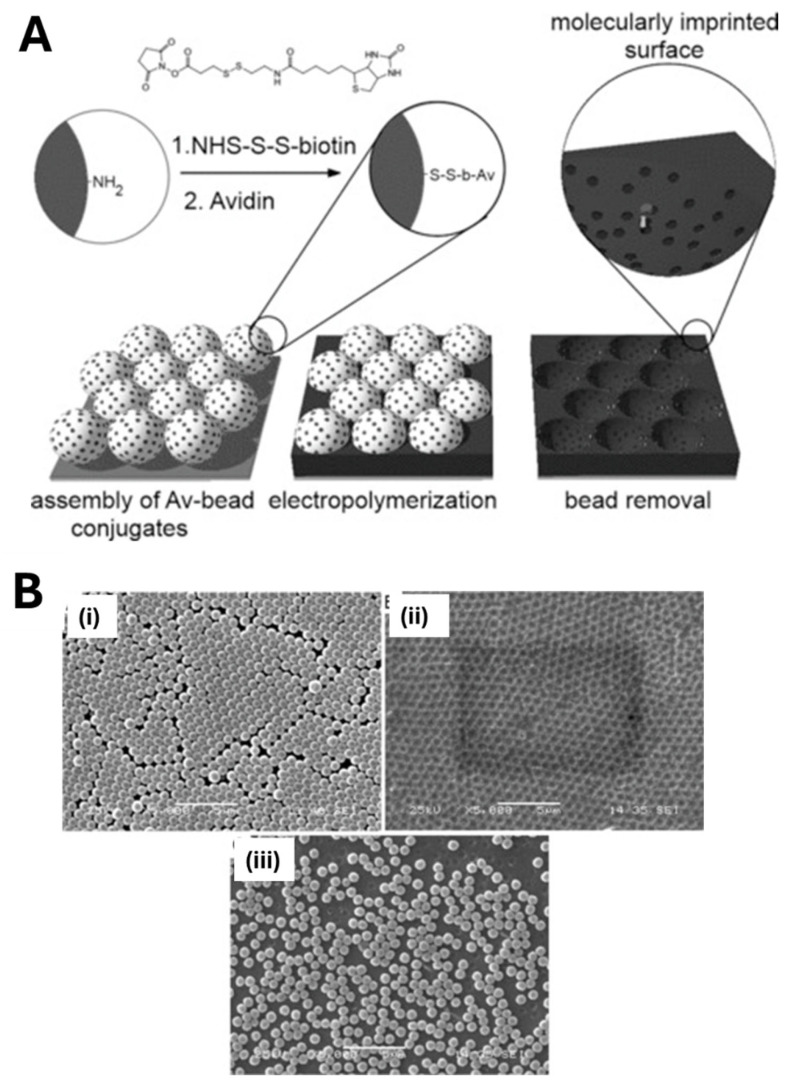
(**A**) Electrosynthesis of surface-imprinted polymer films by nanomolding for selective recognition of avidin. (**B**) SEM images of (**i**) a monolayer of polystyrene beads (ø = 750 nm) drop-cast onto the surface of the quartz crystal resonator; (**ii**) patterned PEDOT/PSS films after electro-polymerization and the removal of the polystyrene beads; (**iii**) partial removal of Av-modified beads revealing both the beads and their imprints in the polymer layer. Adapted with permission from Ref. [76]. Copyright © 2013, Wiley-VCH GmbH.

Pernites et al. [75] introduced an innovative nanomolding approach to fabricate a nanostructured MIP film directly on gold-coated quartz crystal microbalance (QCM) electrodes for enantioselective (+)-norephedrine sensing. First, 500 nm polystyrene (PS) spheres were assembled into a close-packed monolayer on the QCM surface via the Langmuir–Blodgett technique. A terthiophene-based pre-polymer solution containing the norephedrine template was then electro-polymerized into the interstices of the colloidal array. Subsequent removal of the PS by dipping the substrate into THF (for 30 min, twice), followed by template extraction in ethanol/acetonitrile (1:1), yielded a highly ordered porous film studded with molecularly imprinted cavities. This nanopatterned MIP exhibited strong chiral discrimination ability, producing a sensor response to (+)-norephedrine that was three times greater than to its (−)-enantiomer [75]. In a related study, 750 nm PS beads functionalized with an avidin protein target were immobilized onto gold-coated QCM substrates [76]. A poly (3,4-ethylenedioxythiophene)/poly (styrenesulfonate) (PEDOT/PSS) MIP layer was then electro-polymerized to a thickness comparable to the bead radius, filling the voids between beads. After bead removal, the resulting nanostructured film featured uniform imprinted cavities (Figure 6). This imprinted PEDOT/PSS sensor exhibited a 6.5-fold enhancement in avidin binding compared to a non-imprinted control, underscoring the efficacy of the nanomolding strategy coupled with imprinting for high-performance, label-free biomolecular detection.

From the above-reported examples, it emerges that nanomolding strategies offer several compelling advantages for the synthesis of nanostructured MIPs (Table 1). Most notably, precise control over polymer morphology can be achieved with periodic and highly ordered nanostructures, which can enhance, as demonstrated in some cases, analyte accessibility, diffusion kinetics, and signal transduction. According to the results reported in the literature, the scaffold dissolution process, although it represents an additional step in the assembly of the MIP-based sensor, does not affect the batch-to-batch consistency and the reliability of sensor performance. Additionally, since the polymerization occurs within a pre-existing porous material, the reaction is intrinsically confined [2], mitigating challenges associated with controlling free-radical polymerization kinetics: the physical limits of the mold can have a part in “regulating” the polymer growth [77].

Despite these advantages, nanomolding also presents practical challenges [64] (Table 1). Firstly, scaffold removal must be carefully controlled to avoid damaging the imprinted structure or leaving behind residues that can interfere with analyte recognition. Moreover, the polymerization process, often initiated thermally or via UV light, must be optimized to ensure complete infiltration of the mold while preserving the chemical integrity of template molecules. Indeed, biomolecular targets such as proteins, enzymes, antibodies, or nucleic acid strands may not be stable when exposed to organic solvents or harsh conditions (UV light, high temperature) used during polymerization, since denaturation or degradation may occur. Lastly, the mechanical fragility of the resulting nanostructured MIP could limit sensor stability, especially when obtained as free-standing high-aspect-ratio nanostructures.

### 2.2. MIP Nanopatterning

MIP nanopatterning frequently employs photolithography, a versatile, light-driven technique capable of fabricating complex two- and three-dimensional micro- and nanoscale features [64]. In its mask-based form, a photosensitive pre-polymer blend—containing functional monomers, cross-linkers, a porogen, and the template molecule—is first spin-coated onto a solid substrate (e.g., glass, silica, or a metallic electrode). A photomask bearing the desired nanopattern (lines, dots, or lattice arrays) is then precisely aligned over the film, and UV illumination selectively initiates polymerization in the exposed regions. After mask removal and template extraction, this process yields a regular array of nanostructured MIP features with cavities complementary to the target analyte. Maskless variants, such as direct laser writing, offer further flexibility by eliminating the need for physical masks, enabling rapid prototyping of custom patterns and on-the-fly adjustment of feature geometry. This direct deposition approach produces a highly regular array of surface-accessible binding sites with precise control over feature size, spacing, and aspect ratio, thereby maximizing analyte accessibility, enhancing signal transduction, and ensuring reproducible sensor performance.

**Figure 7:**
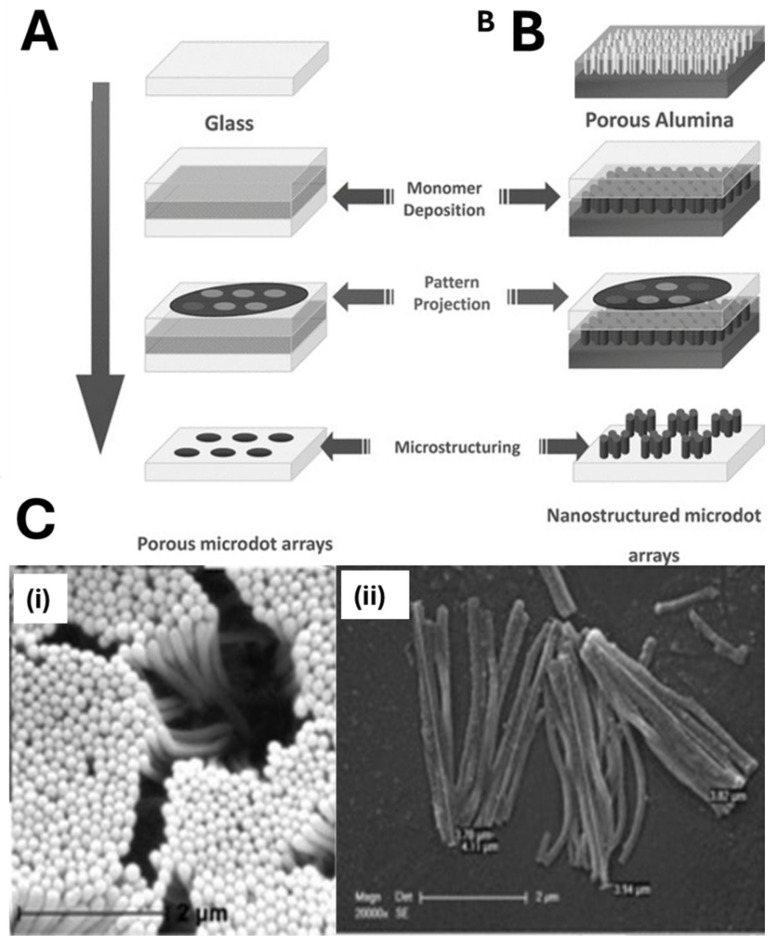
Comparison between MIP deposition on (**A**) flat surface and (**B**) porous alumina combining projection photolithography and nanomolding. (**C**) SEM images of (**i**) polymer nanofilaments and (**ii**) edge of nanofilament dot. Adapted with permission from Ref. [78]. Copyright © 2011, Wiley-VCH GmbH.

Haupt’s group combined microscope projection photolithography with nanomolding to fabricate MIP-based sensors for myoglobin (MYO) and fluorescein [78]. A porous alumina support featuring vertical nanotubular channels (~150 nm in diameter) served as the template. A polymerization mixture (monomers, template, photo-initiator) was first deposited onto this nanostructured substrate. By using a UV projection microscope and a photomask, only selected regions were irradiated, producing MIP nanofilaments exclusively within the masked areas (Figure 7). Employing a 0.5 mm feature photomask and a microscope objective, they fabricated circular sensor “dots” ≈ 70 µm in diameter, each composed of parallel nanofilaments (150 nm diameter, 4 µm length). This localized, high-aspect-ratio architecture greatly increased the surface-to-volume ratio and improved binding site accessibility compared to other porous configurations.

**Figure 8:**
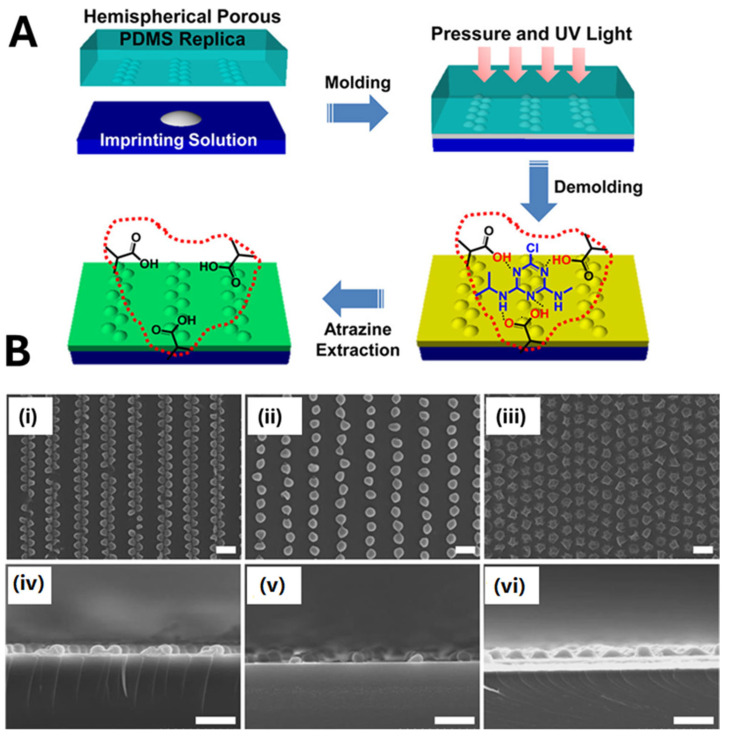
(**A**) Fabrication process of hemispherical MIP films using a PDMS mold; (**B**) SEM images of (**i**) zigzag MIP, (**ii**) linearly isolated discontinuous MIP, and (**iii**) non-close-packed MIP and (**iv**–**vi**) their corresponding cross-sectional images. All scale bars are 1 μm. Adapted with permission from Ref. [80]. Copyright © 2016, American Chemical Society.

Soft lithography, also known as “stamping”, is one of the most widely used approaches for the micro- and nanopatterning of MIPs on flat surfaces. The method typically relies on the use of elastomeric polymeric materials, typically PDMS (polydimethylsiloxane), as a flexible mold or stamp to replicate micro- or nanopatterns on the transducing surface with high resolution and large surface area [39,79]. For example, Yang et al. [80] reported the controlled fabrication of MIP-based colloidal nanoarrays via soft lithography for atrazine sensing using quartz crystal microbalance (QCM) measurements. A gold-coated quartz crystal was functionalized with highly ordered hemispherical MIP nanostructures (Figure 8). To obtain the MIP nanostructures, a PDMS-based nanomold prefilled with the polymerization mixture was pressed onto the gold-coated crystal surface transducer. Then, UV light photo-polymerization (10 min) was applied to produce a two-dimensional hemispherical MIP film on the transducer surface. The removal of the PDMS mold revealed an ordered array of MIP hemispheres (Figure 8A). The approach is very interesting, since it can be used to tune the size, shape, and distribution of the MIP nanostructures on the transducer surface (Figure 8B). Compared to a planar MIP film, this hemispherical 2D architecture yielded a 6.5-fold increase in sensitivity, underscoring the efficacy of nanomolding for QCM-based sensing [80]. However, incomplete demolding or surface contamination may occlude binding cavities or alter polymer chemistry, necessitating a careful optimization of mold release and post-processing washes to preserve the molecular recognition properties of the imprinted film.

**Figure 9:**
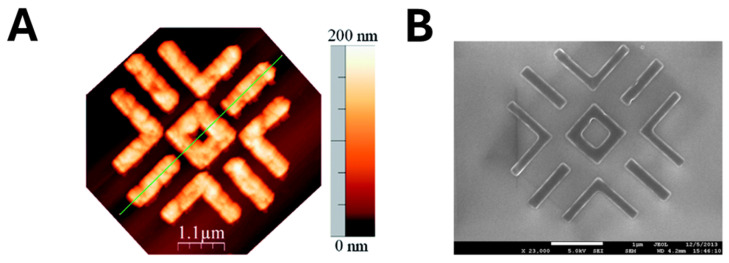
(**A**) AFM image of an MIP nanostructure fabricated by EBL. Electron radiation dose: 750 μC cm^−2^ (positive tone resist); (**B**) SEM micrographs corresponding to an MIP nanostructure fabricated by e-beam direct writing at an electron radiation dose of 750 μC cm^−2^ (positive tone resist). Adapted with permission from Ref. [81]. Copyright © 2014, American Chemical Society.

Electron beam lithography (EBL) partially solves these issues, as it is a mask-free, non-contact nanopatterning technique with nanometer precision, eliminating the risk of mold-related contamination. In a landmark study by Moreno-Bondi’s group [81], a single linear co-polymer, poly (methacrylic acid-co-2-methacrylamidoethyl methacrylate) [P (MAA-co-MAAEMA)], served both as a positive-tone electron beam resist and as the imprinting matrix for rhodamine 123 (R123) recognition. A viscous solution of the co-polymer, pre-loaded with R123, was spin-coated onto a piranha-cleaned Si (100) wafer. During exposure, the focused electron beam induced cross-linking of the polymer chains only in the irradiated regions, rendering them insoluble in the subsequent developer bath. In contrast, the unexposed polymer dissolved away, leaving behind nanometer-precise polymer features that retained the embedded template. Direct writing with a 750 µC cm^−2^ electron dose produced ultrafine MIP features with exceptional spatial accuracy (Figure 9). Finally, solvent extraction removed R123 from the cross-linked domains, revealing high-fidelity, surface-accessible cavities complementary to the dye. This approach seamlessly integrates pattern definition, template entrapment, and cavity formation into a single EBL workflow, delivering ultrafine MIP nanostructures with exceptional spatial accuracy.

**Figure 10:**
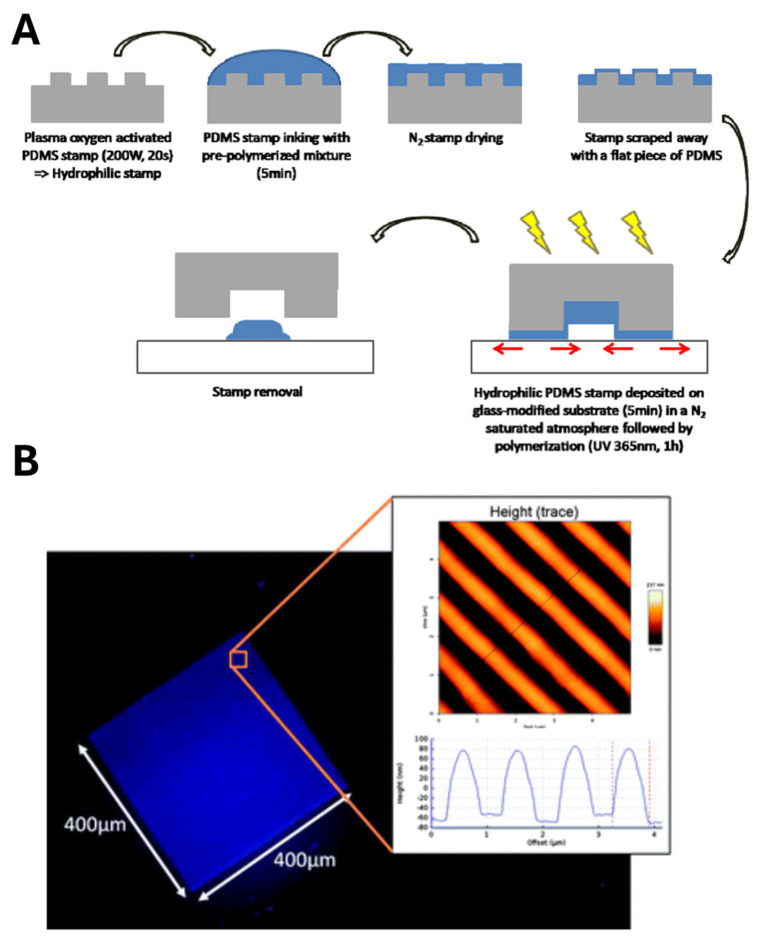
(**A**) Schematic representation of the transfer process involved in the MIP nanopatterning using a hydrophilic PDMS stamp. (**B**) Dark-field microscopy image of an MIP nanopatterned by soft lithography. Inset: AFM topography scan of the MIP nanolayer. Adapted with permission from Ref. [82]. Copyright © 2010, American Chemical Society.

In another notable demonstration, Lalo et al. [82] successfully nanopatterned MIP films on glass substrates for the fluorescent detection of dansyl-L-phenylalanine. The process began by fabricating a silicon master via electron beam lithography. A PDMS mold was cast from this master, cured at 80 °C for seven hours, then rendered hydrophilic by oxygen plasma treatment (Figure 10A). The MIP pre-polymer mixture—containing a functional monomer, cross-linker, initiator, porogen and template—was drop-cast onto the patterned PDMS, with excess removed under a gentle N_2_ stream. A flat glass slide was then placed atop the mold, and UV irradiation (365 nm) for one hour induced polymerization. Peeling away the PDMS revealed three-dimensional MIP nanostructures measuring approximately 400 µm in length, 660 ± 10 nm in width, and 140 ± 5 nm in height, with sharp, smooth features (Figure 10A). Upon exposure to dansyl-L-phenylalanine, these nanoreceptors exhibited a pronounced increase in fluorescence, significantly outperforming non-imprinted controls and confirming both the fidelity of the imprinting process and the high binding capacity of the nanopatterned MIPs.

Similarly, Forchheimer et al. [83] utilized nanoimprinting lithography to obtain propranolol-imprinted nanostructures on a glass substrate with a height of 250 nm and 100 µm width. Nanopatterned Si wafers were used as a stamp, while UV light was utilized for polymerization. The MIP binding analysis was performed by radioligand binding analysis and a 3.5-fold higher response of MIP was recorded compared to NIP nanostructures, proving the imprinting strategy.

**Figure 11:**
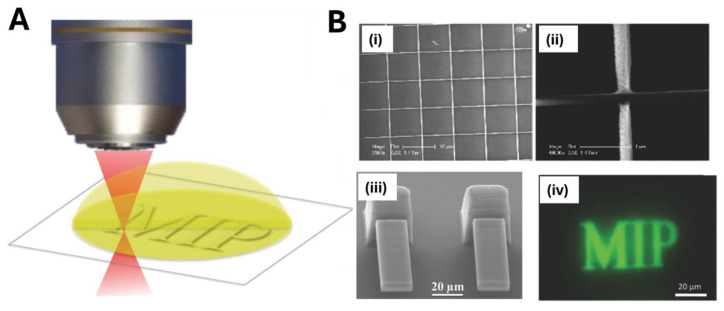
(**A**) Schematized representation of the direct laser writing of MIPs by displacing an NIR femtosecond laser focused beam inside the MIP precursor solution. (**B**) SEM images of MIP structures generated by TPS: (**i**) and (**ii**) wires, (**iii**) cantilevers, (**iv**) fluorescence microscopy after binding of a fluorescent derivative of the template (dansyl-l-Phe). Adapted with permission from Ref. [84]. Copyright © 2016, Wiley-VCH GmbH.

Two-photon stereolithography (TPSL) is another fascinating technique that enables the direct fabrication of three-dimensional nanostructures on flat surfaces utilizing a photo-polymerizable material [64]. TPL relies on a two-photon polymerization (TPP) process that involves the sensitization of a photo-initiator via two-photon excitation and the subsequent cross-linking of a monomer/oligomer (polymerizable resin or photo-resist), using an intense pulsed laser beam. TPSL offers unparalleled freedom to “print” three-dimensional MIP architectures directly onto planar substrates. In a representative study [84], Gomez et al. applied this approach to “print” both micro- and nanoscale MIP architectures on glass: using a 40× objective (10 ms, 15 mW), they fabricated 20 × 60 µm cantilever sensors (5 µm thick), and with a 100× objective (10 ms, 10 mW) they produced crossed line features as narrow as 250 nm. For MIP synthesis, the polymerization mixture, containing monomers, the target, and Lucirin-TPO as the photo-initiator, was placed on a glass substrate. Then, the photo-polymerization was performed using a femtosecond laser source combined with an objective magnification system (Figure 11). After laser exposure, unpolymerized resin was removed in ethanol and the template extracted with a methanol–acetic acid wash, yielding functional MIP micro- and submicro-structures used for label-free detection of the target.

Despite the fascinating nanostructures obtained by this approach, some limitations can be highlighted [85]. Since, when using TPSL, a 3D object is built up point-by-point, overall throughput is low, making large-area or high-density patterning time-consuming and costly. Additionally, the process relies on specialized two-photon active resins—which are not yet commercially standardized—so each laboratory must formulate and optimize its own pre-polymer mixture, compromising reproducibility. Moreover, the polymerizable volume is strictly confined to the focal spot (on the order of femtoliters), so scaling-up is highly demanding. The requirement for laser intensities above a precise two-photon threshold creates a narrow “process window”: slight deviations in power or focus yield incomplete curing or excessive cross-linking, distorting feature dimensions or degrading embedded templates. Until advances in resin chemistry, multi-beam writing strategies, and high-speed scanning are achieved, TPSL remains an extraordinary yet niche tool for prototyping MIP nanostructures rather than a scalable route to mass-produced sensors.

In nanopatterning techniques, the required use of cleanroom facilities, expensive instrumentation, and multi-step fabrication processes represent possible limitations significantly increasing cost and complexity of the nanostructured MIP assembly. Moreover, the use of harsh chemicals or solvents during development and lift-off steps can also damage sensitive substrates or alter the properties of the obtained MIP. In addition, the achievable patterning area is often constrained by the resolution and throughput of the technique employed, making large-scale or high-throughput sensor manufacturing challenging (Table 1).

## 3. Direct Integration of MIPs with Nanotransducers

### 3.1. MIP Electro-Polymerization

The direct integration of MIPs on nanotransducers via electro-polymerization leverages the intrinsic ability of electro-polymerizable monomers to form ultrathin and conformal films directly on conductive surfaces under an applied potential, typically through an oxidative polymerization process [18,59]. Starting from a pre-polymerization solution containing the functional monomer, the template molecule, and the supporting electrolyte in contact with a nanostructured electrode, the obtained MIP film is characterized by exceptional adhesion to the electrode surface, achieved without the need for chemical coupling agents or postdeposition processing [18,86,87,88], and by a finely tunable thickness, enabled by the control of the total passed charge, which guarantees high reproducible MIP deposition even on high-aspect-ratio nanostructures.

**Figure 12:**
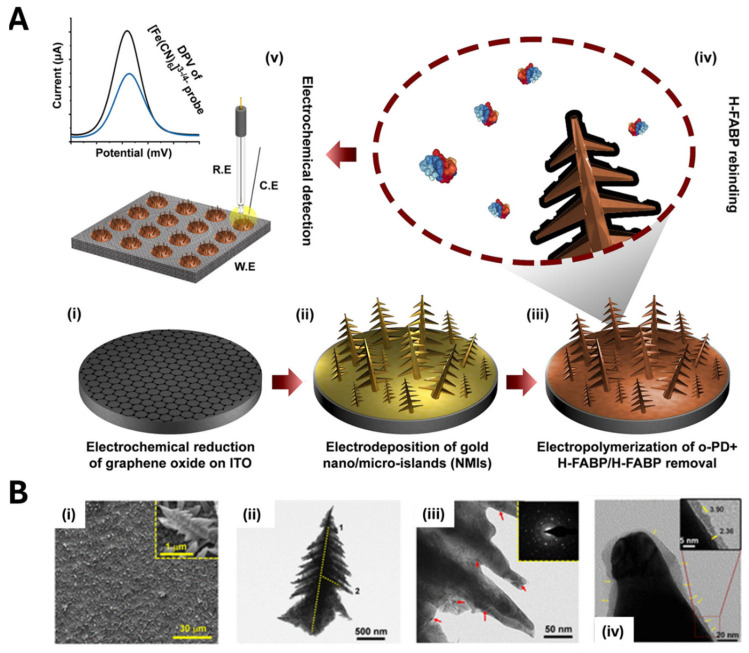
(**A**) Schematic illustration of NMI deposition and MIP fabrication by electro-polymerization on NMI-modified nanostructured electrode for H-FABP detection. (**i**) Modification of indium–titanium oxide (ITO) surface with GO; (**ii**) electrodeposition of NMIs on modified ITO electrode; (**iii**) electrochemical deposition of MIP on NMIs; (**iv**) theoretical representation of MIP cavities on the NMI surface; (**v**) changes in readout upon H-FABP binding with MIP. (**B**) Microstructural properties of the modified electrodes. SEM images of the (**i**) NMIs functionalized electrode (inset: high-magnification SEM image of NMIs); (**ii**,**iii**) TEM images of a gold shrub-like structure (arrows display imperfections, and the inset shows the corresponding SAED pattern); (**iv**) TEM image of the MIP layer on NMIs. Adapted with permission from Ref. [90]. Copyright © 2021, American Chemical Society.

Among these, gold nanostructures have long been favored for MIP-based electrochemical sensors due to their high conductivity, tunable sizes and shapes, and a surface chemistry that readily supports functionalization [89]. Sanati et al. [90] modified an indium–tin oxide (ITO) electrode with reduced graphene oxide (rGO) to create a rough, high-surface-area scaffold, onto which they electrochemically deposited gold nano- and micro-islands (NMIs) via chronoamperometry (Figure 12A). SEM and TEM images revealed free-standing, shrub-like structures (2.2 μm long, 50–300 nm wide) (Figure 12B). Placing the electrode in a solution containing o-phenylenediamine (o-PD) as the monomer and the heart-type fatty acid binding protein (H-FABP) as the template, and employing cyclic voltammetry, an MIP nanolayer (approximately 5.5 nm thick) was obtained around the NMIs. Compared to flat electrodes, the NMI-based sensor showed higher performance, achieving an LOD of 2.29 fg·mL^−1^, and rapid rebinding kinetics (30 s), due to enhanced analyte diffusion and solvent permeation within the nano-islands.

Nanoporous gold (NPG) similarly provides an interconnected, conductive framework fabricated by alloying gold with active metals such as silver, zinc, copper, aluminum, etc., that are removed by dealloying, leading to the generation of nanoporous gold layers on the electrode surface. Cheng et al. fabricated NPG on glassy carbon electrodes (GCEs) by alloying gold with silver and then dealloying in nitric acid. After anchoring the target analyte to the porous surface via aminothiophenol coupling, they electro-polymerized MIP receptors for 3-monochloropropane-1,2-diol detection by cycling the potential between −0.3 V and 1.2 V for 20 CV cycles; SEM confirmed the deposition of an ultrathin and conformal MIP coating on NPG [91].

In another contribution [92], Li et al. extended this approach to warfarin sensing, comparing four monomers (resorcinol, dopamine, o-aminophenol, phenylenediamine) and selecting resorcinol for its superior binding efficiency; the resulting MIP film (≈10 nm thick) yielded highly sensitive detection on NPG. Song et al. [93] functionalized a solid Au-Ag nanoporous alloy microrod (NPAMR), facilely prepared by dealloying of a smooth Au-Ag alloy microrod, and used it as an electrode by electrodepositing an *o*-PD-based MIP layer in the presence of MNZ as the template. The functionalization of NPAMR with MIP layers offered great features in terms of surface area and binding site density, as demonstrated by the low detection limits reached.

NPG could be integrated with further nanomaterials to improve its features. For example, transition metal carbides such as Ti_3_C_2_Tx MXenes have been integrated with NPG to further boost performance. Zhang et al. [47] dispersed MXene in a chitosan–acetic acid solution and drop-cast it onto NPG and then evaporated the solvent under infrared light. Electro-polymerization of o-aminothiophenol and resorcinol in the presence of thiabendazole produced a uniform MIP layer, demonstrating the versatility of combining multiple nanomaterials to amplify conductivity and binding site density.

**Figure 13:**
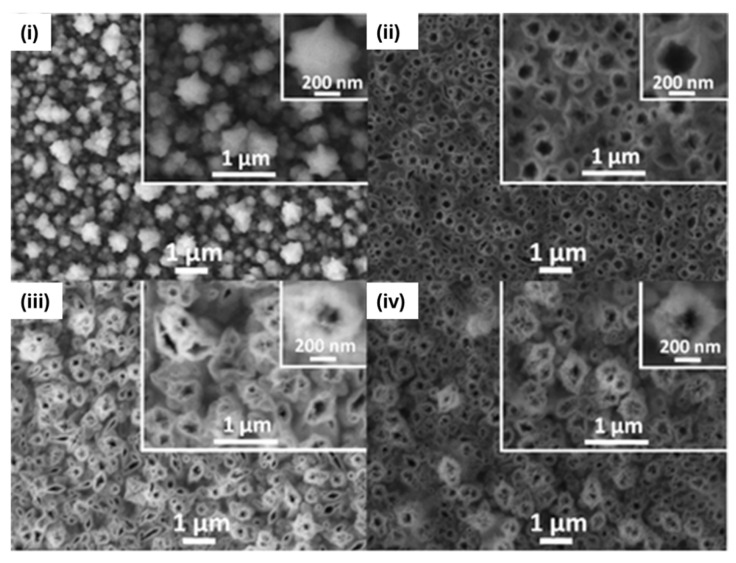
SEM micrographs of Cu–Ni alloy (**i**), NPNi (**ii**), and MIP/NPNi before (**iii**) and after (**iv**) extraction of MNZ. Adapted with permission from Ref. [48]. Copyright © 2015, American Chemical Society.

Electro-polymerization has also been employed on other conductive scaffolds. For instance, a 3D nanoporous nickel (NPNi)/MIP composite was prepared by electrodepositing Ni/Cu alloy onto a GCE and then dealloying to yield nanosized (100–200 nm) flower-like structures (Figure 13). Subsequent cyclic voltammetry (150 CV cycles) of o-PD in the presence of a metronidazole (MZN) template produced a conformal MIP layer. The sensor achieved a remarkable sensitivity of 2 × 10^−14^ M and excellent selectivity and stability over 30 days. MIP deposition on NPNi was monitored by checking its material features, namely wall thickness and pore size, together with electrochemical characterization [48].

**Figure 14:**
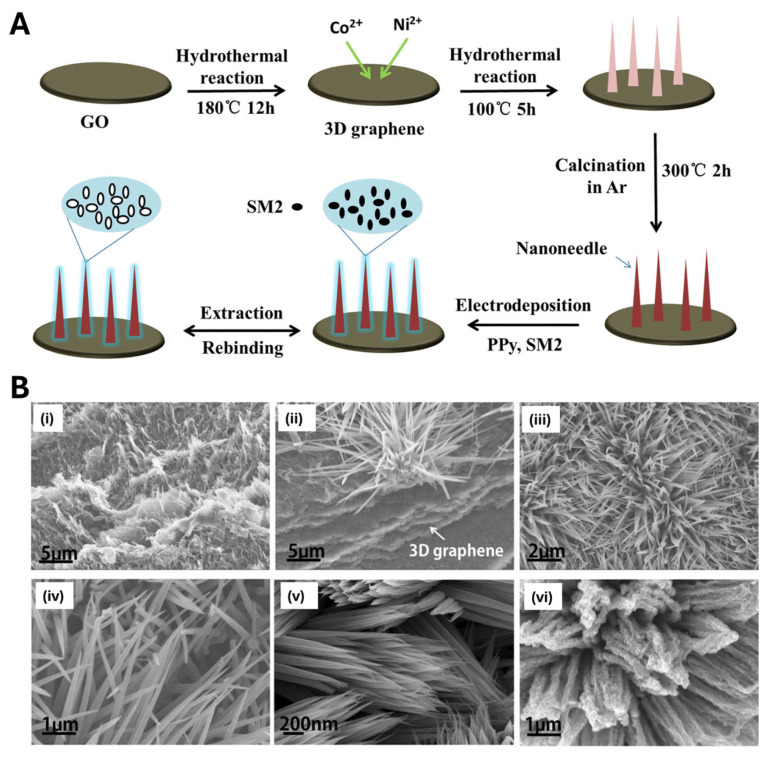
(**A**) Structuring free-standing NiCo_2_O_4_/3D nanoneedles on GO along with electrodeposition of MIP for SM_2_ sensing. (**B**) SEM images of (**i**) graphene; (**i**–**v**) NiCo_2_O_4_ nanoneedles/3D graphene; and (**vi**) MIP/NiCo_2_O_4_/3D graphene. Adapted with permission from Ref. [94]. Copyright © 2018, Springer Nature.

In another example [94], Wei et al. decorated 3D graphene oxide with NiCo_2_O_4_ nanoneedles—produced via a two-step hydrothermal route—and then electro-polymerized a PPy-based MIP for sulfadimidine detection in milk. The MIP layer was deposited onto the nanostructured substrate by cyclic voltammetry (10 cycles from –0.5 to +0.15 V at 100 mV/s), yielding a conformal film layer (approximately 7 nm thick) on the nanoneedle surface (Figure 14). By combining this architecture with the selective recognition capabilities of a conductive polypyrrole-based MIP, the authors achieved an ultrasensitive sensor (LOD = 0.169 ng/mL) that performs reliably in real samples [94].

Recent innovations have extended MIP electro-polymerization into other nanoformats, designed for further extending the application of the resulting sensors, for example, in biomedical fields, such as transdermal microneedle arrays (MNAs). Oliveira et al. [95] molded polycarbonate MNAs, metallized them to define the working, counter, and reference electrodes, and then electro-polymerized an MIP for interleukin-6 detection. MIP receptors were obtained in two steps involving electro-polymerization of the monomer 3-aminophenylboronic acid (APBA) followed by subsequent protein removal. In the first step, a solution consisting of APBA (5 mM) and IL-6 (10 μg/mL) in PBS buffer (pH = 7.4) was used to assemble the MIP layer by electro-polymerization on one of the MNAs and designated as the MIP array. This was carried out by CV (−0.2 to 1.0 V, 15 cycles, 0.02 V/s). Their MIP-PDA MNAs detected IL-6 down to 1 pg/mL in artificial interstitial fluid, demonstrating the potential for minimally invasive, pain-free biomarker monitoring.

In addition, electro-polymerization has been paired with photoelectrochemical platforms [51]. Titanium dioxide (TiO_2_) has attracted growing interest in photoelectrochemical sensing due to its unique semiconductor properties. In recent years, researchers have combined TiO_2_ nanostructures with molecularly imprinted polymers (MIPs) to create hybrid photoelectrochemical sensors. For example, Shi et al. [51] fabricated a sensor for 2,4-dichlorophenoxyacetic acid (2,4-D) on TiO_2_ nanotube arrays produced by anodic oxidation of titanium foil. They electro-polymerized polypyrrole (PPy) around the nanotubes by cycling the potential between –0.2 and +1.0 V at 50 mV s^−1^ for ten cycles in a solution containing pyrrole monomer and the 2,4-D template. Under irradiation, the PPy@TiO_2_ nanotubes imprinted with 2,4-D exhibited a significantly higher photocurrent than both non-imprinted PPy@TiO_2_ and bare TiO_2_ nanotubes, which was due to the MIP’s ability to selectively bind and concentrate 2,4-D at the electrode surface, promoting its photocatalytic oxidation.

In a related study [53], Au-decorated TiO_2_ nanotubes (60 nm diameter) were electrodeposited, followed by o-PD electro-polymerization, to form an MIP that not only served as a selective recognition element but also improved charge separation and photocurrent generation under UV; this tandem architecture minimized UV-induced damage to templates and amplified sensing performance.

Electro-polymerization thus offers a powerful route to deposit MIP thin films or nanostructured transducers (nanofilaments, nanorods, nanopores) under different conditions (namely, galvanostatic, potentiostatic, or potentiodynamic control) in mild, ambient conditions [13], affording rigorous control over polymer thickness, excellent reproducibility, and rapid reaction rates. Anyhow, the need for a conductive substrate partially limits its application (Table 1). Also, the possible modification of the template redox state—especially if it is electroactive under the applied conditions—during electro-polymerization should be considered. Importantly, issues deriving from monomer diffusion, especially in high-aspect-ratio nanostructures, could lead to uneven MIP layer deposition on the nanostructured transducer, possibly reducing sensor performance. Finally, gas evolution (oxygen or hydrogen) during electrochemical polymerization may introduce unwanted defects or porosity into the film.

### 3.2. Dopamine Self-Polymerization

The dopamine self-polymerization approach has emerged as a popular technique for the assembly of MIP-based sensors, leveraging its well-known simplicity and versatility [12,96,97,98]. Generally, dopamine oxidizes and self-polymerizes in alkaline solutions to form a stable polydopamine (PDA) coating on a wide range of substrates [12,96,97,98]. The process can be further accelerated by introducing oxidizing agents such as ammonium persulfate [99,100,101], offering a facile and equipment-free route to surface functionalization.

**Figure 15:**
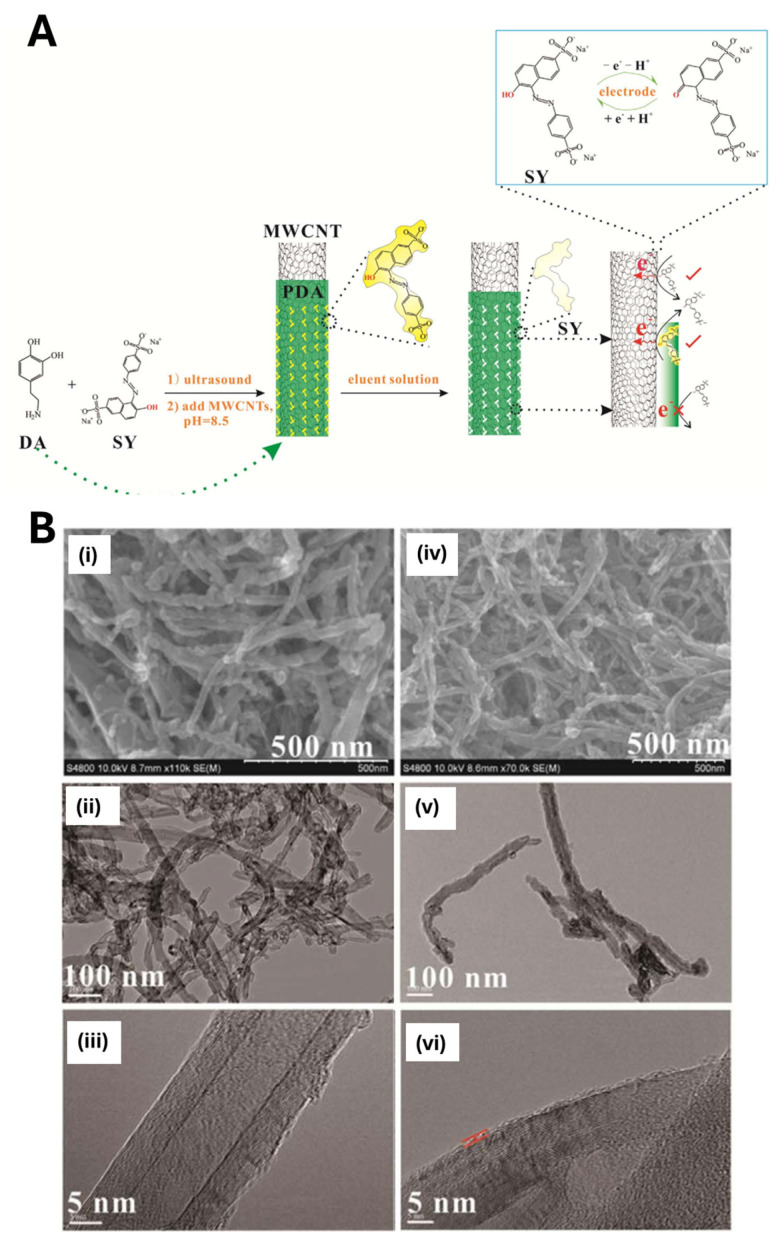
(**A**) The preparation of MWCNT@MIP-PDA and the mechanism of the electrochemical redox processes of SY. (**B**) SEM (upper), TEM (middle), and HRTEM (lower) images of (**i**–**iii**) MWCNTs, MWCNT@MIP-PDA (**iv**–**vi**). Adapted with permission from Ref. [102]. Copyright © 2018, Elsevier.

Although PDA-based MIPs have been successfully integrated with nanoparticles, carbon dots, and quantum dots, few studies have investigated PDA coatings on more complex nanotransducers. For example [102], Yin and colleagues developed an electrochemical sensor for the food dye Sunset Yellow (SY) by coating acid-treated multiwalled carbon nanotubes (MWCNTs) with a thin PDA–MIP film. They dispersed MWCNTs in a Tris buffer (pH 8.5) containing dopamine and SY; under these conditions, dopamine self-polymerized around the nanotubes, entrapping SY within the PDA matrix (Figure 15). After washing with an acid–ethanol mixture to remove the template, they deposited the MWCNT@PDA–MIP composite onto a glassy carbon electrode by drop-casting. Once dry, the electrode was ready for the electrochemical measurements: SY oxidation produces a current peak proportional to its concentration. The combination of MWCNTs’ excellent conductivity and high surface area with a conformal, one-step aqueous PDA coating yielded a highly sensitive SY sensor with rapid response and minimal preparation complexity.

**Figure 16:**
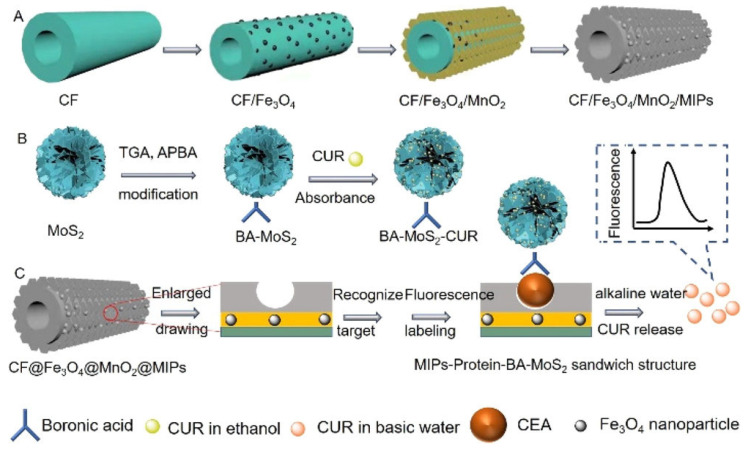
General illustration of (**A**) fabricating PDa-based CF/Fe_3_O_4_/MnO_2_/MIP; (**B**) synthesis of BA-modified MoS_2_-CR fluorescent tag; (**C**) detection process of MIP-based fluorescent sensor. Reprinted with permission from Ref. [103]. Copyright © 2022, American Chemical Society.

Lu et al. [103] developed a “sandwich”-type fluorescence sensor for ultrasensitive detection of carcinoembryonic antigen (CEA) by combining hierarchically structured carbon nanofibers (CNFs), magnetic/oxidic coatings (Fe_3_O_4_ and MnO_2_), and a thin molecularly imprinted polydopamine layer (Figure 16). Carbon nanofibers are first grown by chemical vapor-phase deposition (CVD) and then coated with superparamagnetic Fe_3_O_4_ nanoparticles via a solvothermal route. On top of those, ultrathin MnO_2_ nanosheets are deposited in situ. The Fe_3_O_4_ imparts facile magnetic separation, while MnO_2_ serves as an efficient fluorescence quencher. This hierarchical CNF@Fe_3_O_4_@MnO_2_ composite thus provides a high-surface-area backbone and strong background quenching to minimize nonspecific fluorescence. A dopamine polymerization mixture—containing a dopamine monomer and CEA as the template—is introduced onto the CNF@Fe_3_O_4_@MnO_2_ surface. Under weak alkaline conditions, polydopamine self-polymerizes around the CEA, creating a thin, conformal MIP layer upon target removal. The obtained CNF@Fe_3_O_4_@MnO_2_/MIPs can specifically absorb the target CEA from complex samples, which can then be specifically labeled with boronic acid-modified MoS_2_-curcumin nanotags (BA-MoS_2_-CUR). Under alkaline conditions, curcumin was detached from MoS_2_ and used as a fluorescence reporter molecule. The fluorescence increased with CEA concentration in the analyzed samples due to the affinity of BA-MoS_2_-CUR with the target: curcumin release within the solution was proportional to CEA concentration, recording a related increase in fluorescence coming from the released curcumin.

Although dopamine polymerization requires no external catalysts or extreme conditions, it still poses some challenges when functionalizing nanotransducers for MIP-based nanosensors (Table 1). First, achieving precise control over the PDA film thickness is not easy, as self-polymerization kinetics are highly sensitive to operative conditions such as pH, temperature, dissolved oxygen, and reaction time [12,104]. The lack of precise control over polydopamine polymerization frequently causes the aggregation of dopamine micro- and nanoparticles [12,104]. This, in turn, leads to increased surface roughness, spatial variability in signal across transducers, and low reproducibility, ultimately hindering consistent polymer deposition—particularly on nanostructured surfaces with a high aspect ratio. Moreover, although PDA deposition is generally substrate-independent, the alkaline, oxidative environment used to drive polymerization can denature sensitive biomolecules templates.

### 3.3. Localized Polymerization

In the localized polymerization approach, MIP films are synthesized exclusively in the immediate vicinity of the nanotransducer surface by confining the polymerization initiation event. This spatial confinement enables precise control over polymer growth, ensuring intimate contact between the recognition sites and the transducing interface [105,106,107,108].

Two main strategies are typically employed to achieve such localization: (i) stimulus-confined polymerization, where a focused light stimulus, restricted to a selected area, is used to selectively initiate polymerization at targeted nanoscale features, similarly to what happens for the light confinement used in other techniques, such as electron beam lithography (EBL) and two-photon stereolithography (TPSL), enabling highly localized MIP deposition [64]; and (ii) surface-initiated polymerization, in which initiators are grafted onto the transducer surface, and controlled polymerization occurs selectively in the functionalized regions.

Since initiation is spatially restricted in both cases, the polymer grows as an ultrathin, conformal shell that mirrors the underlying nanostructure. Subsequent template extraction reveals imprinted cavities precisely positioned to exploit the enhanced electric field, plasmonic hotspot, optical sensitivity, or electroactivity of the nanotransducer. This one-step, mask-free technique achieves exceptional spatial precision (down to tens of nanometers), yielding ultrathin MIP layers that preserve the intrinsic properties of the transducer at the nanoscale, allowing for rapid and sensitive target detection.

**Figure 17:**
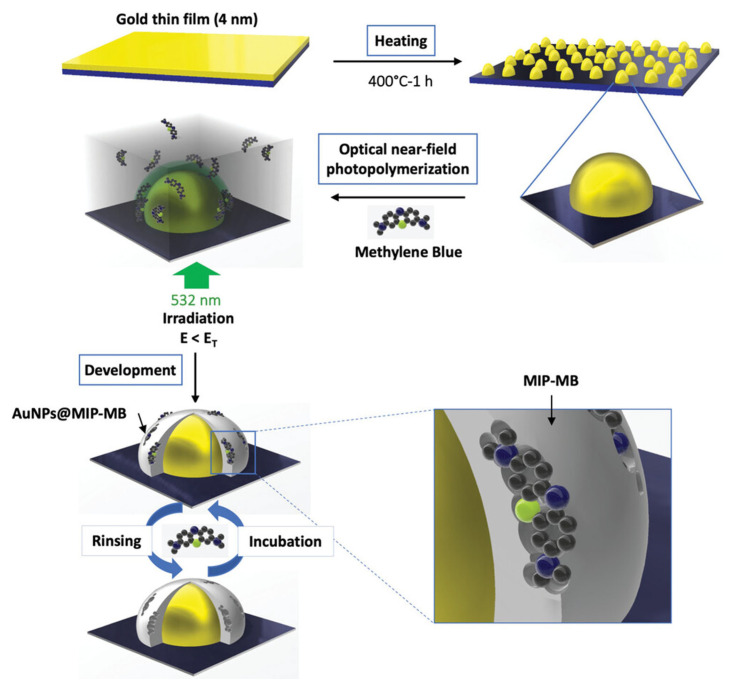
AuNP synthesis by thermal de-wetting (preparation of glass slide, deposition of 4 nm gold layer, heating of the gold layer at 400 °C for 1 h to obtain AuNPs) and subsequent deposition of MIP by near-field photo-polymerization, irradiating with a green light (532 nm). Reprinted with permission from Ref. [61]. Copyright © 2023, Wiley-VCH GmbH.

In a very interesting work [61], Haupt’s group employed optical near-field photo-polymerization (ONFP) of MIPs on gold nanoparticles (AuNPs) grafted onto a glass slide (Figure 17). ONFP is a nanoscale photofabrication technique that exploits a localized optical near-field to initiate photo-polymerization beyond the diffraction limit of light [109,110]. By confining the excitation to tiny volumes, typically using sharp tips, nanoapertures, or plasmonic structures, ONFP enables direct writing of polymer structures with spatial resolution below 100 nm, overcoming the ~200–300 nm limit of conventional far-field optics. In [61], AuNPs were first formed by thermal de-wetting of a 4 nm gold film at 400 °C, yielding a uniform nanoparticle distribution. Later, a drop of the polymerization mixture was deposited on the substrate containing AuNPs and covered by a glass coverslip. Then, the photo-polymerization was performed using a laser emitting at 532 nm with a power of 50 mW cm^−2^. For the deposition of an ultrathin polymeric shell (~2 nm) around the resonant AuNPs, the irradiation dose (*E*) was equal to 90% of the irradiation threshold dose (*E*_T_). To determine the *E*_T_, defined as the light dose required to obtain polymer on glass slides, a drop of the formulation was placed between two glass coverslips and irradiated with a 532 nm continuous laser. During the polymerization, titanocene served as the photo-initiator, MAA and EGDMA as the monomer and cross-linker, and pentaerythritol triacrylate (PETA) as supporting trifunctional cross-linker. The resulting AuNP@MIP hybrids on glass substrates functioned as sensitive platforms for localized surface plasmon resonance (LSPR) and surface-enhanced Raman spectroscopy (SERS), demonstrating excellent specificity toward methylene blue (MB) with rapid response times (10 min).

Localized MIP photo-polymerization on a nanotransducer can also be achieved by exploiting controlled radical polymerization approaches to confine imprinting to nanoscopic regions. One strategy is surface-initiated photo-iniferter polymerization, in which an iniferter agent, typically bearing a thiocarbonylthio group, is covalently grafted to the nanotransducer surface. The iniferter is an agent that acts as initiator, transfer agent, and terminator during the polymerization procedure [64,111,112]. Upon irradiation (UV or visible light), the iniferter cleaves to generate radicals, which initiate polymer growth only at the surface and then reversibly deactivate to maintain control over the polymer chain length and architecture. This yields an ultrathin MIP layer (often <10 nm) with narrow molecular weight distributions and highly uniform imprint sites.

**Figure 18:**
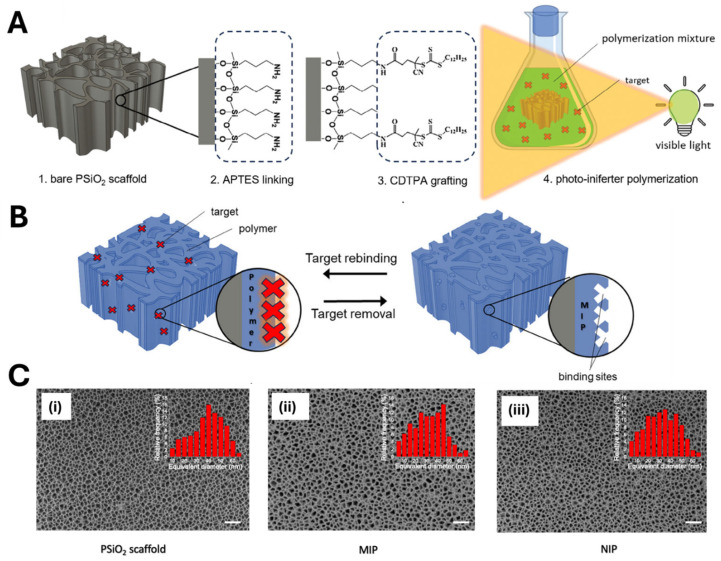
(**A**) MIP deposition on PSiO_2_ scaffold by photo-iniferter polymerization, which includes the modification of the bare PSiO_2_ (**1**) with APTES (**2**), CDTPA grafting through a coupling reaction (**3**), and photo-iniferter polymerization (**4**) to obtain a poly (MAA-co-EGDMA)-based MIP film. (**B**) Schematic representation of the reversible interaction of propranolol (red crosses) with MIP on PSiO_2_ scaffold. (**C**) SEM images of (**i**) the bare PSiO_2_ scaffold and (**ii**) MIP- and (**iii**) NIP-functionalized PSiO_2_ samples. Adapted with permission from Ref. [45]. Copyright © 2025, Wiley-VCH GmbH.

Very recently, visible-light photo-iniferter polymerization has been proposed to achieve the direct integration of MIP receptors into nanoporous silicon (PSiO_2_) scaffolds, used as optical transducers [45]. Since PSiO_2_ transducer has a complex honeycomb geometry (≈50 nm pores, 4 mm length), uniform functionalization required anchoring the photo-iniferter (CDTPA: 4-cyano-4-[(dodecylsulfanylthiocarbonyl) sulfanyl]pentanoic acid) onto the silica surface via amine-mediated coupling (Figure 18). Indeed, grafting the photo-iniferter to the nanostructured surface has two main advantages: first, it provides high control over the polymer growth and adhesion within the nanoporous material; second, it prevents the growth of a free oligo/polymer in the polymerization solution, thus avoiding the possible blocking of nanopores. After iniferter grafting, the devices were then immersed in a polymerization mixture containing templates (propranolol or atenolol), MAA, and EGDMA and exposed to a 525 nm LED. Under optimized conditions (5 h irradiation), a homogeneous MIP thin layer (3.7 ± 0.6 nm) formed throughout the porous scaffold. The resulting sensors detected atenolol or propranolol at micromolar concentrations in tap water.

**Figure 19:**
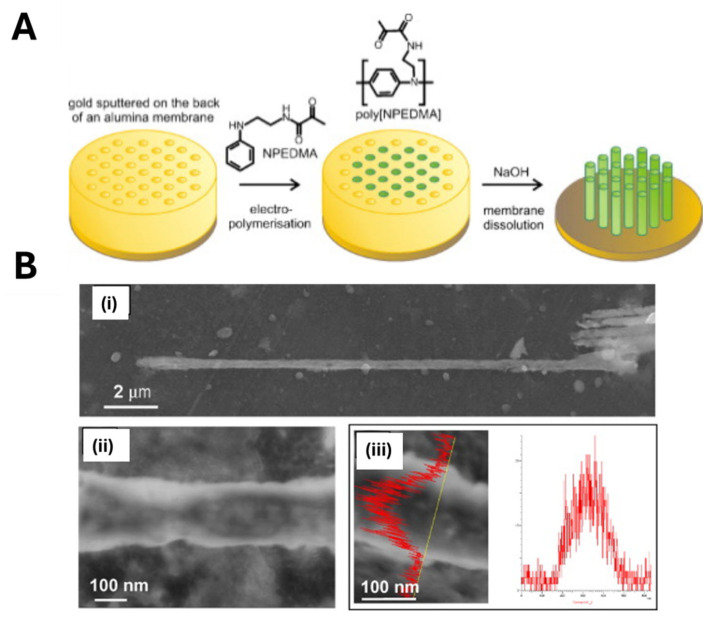
(**A**) Scheme of the template synthesis of poly (NPEDMA) nanostructures. (**B**) SEM images of (**i**) single poly (NPEDMA), several microns long; (**ii**) detail of a single poly (NPEDMA) nanotube; and (**iii**) carbon mapping profile along the section of the nanostructure obtain by Energy-Dispersive X-ray (EDX) spectroscopy analysis. Adapted with permission from Ref. [113]. Copyright © 2010, Elsevier.

Marrazza’s group utilized photo-iniferter polymerization of poly (*N*-phenylethylene diamine methacrylamide) (poly (NPEDMA)) nanofilaments for the electrochemical sensing of catechol [113]. First, vertically aligned conductive poly (NPEDMA) nanofilaments (150 nm diameter, 50 µm length) were obtained on a gold electrode surface by the nanomolding technique using a nanoporous alumina membrane as a sacrificial mold (Figure 19A). The poly (NPEDMA) nanofilaments were obtained performing the electro-polymerization of a solution containing *N*-phenylethylene diamine methacrylamide (NPEDMA), used as monomer, bearing both aniline and methacrylamide moieties. Next, a diethyl dithiocarbamic benzyl ester iniferter was immobilized onto the poly (NPEDMA) surface by UV-induced photochemical treatment. The electrode was then immersed in a polymerization mixture containing urocanic acid ethyl ester (monomer), catechol (template), and EGDMA (cross-linker), and irradiated with UV light for 30 min. Photo-iniferter polymerization ensured the controlled deposition of MIP receptors, preserving the poly (NPEDMA) nanofilament dimensions (Figure 19) without compromising their conductive properties. The resulting electrochemical MIP sensor exhibited a tenfold higher sensitivity toward catechol compared to structurally similar interferents.

To achieve tight control over nanoscale MIP coatings, researchers have also used other controlled/living radical polymerization methods, such as surface-initiated atom transfer radical polymerization (SI-ATRP) [114,115] or surface-initiated reversible addition-fragmentation chain transfer polymerization (SI-RAFT) [116,117]. Generally, SI-ATRP is applied by grafting an alkyl halide initiator on the transducer surface, which is reversibly activated by a metal-catalyzed redox cycle, typically involving a complex formation with a ligand, at elevated temperatures (50–150 °C). Upon oxidation of the metal center, the C-X bond of the initiator cleaves to form carbon radicals that drive chain propagation, leading to polymer deposition on the transducer surface [118,119]. As an example, Oh et al. [120] constructed an MIP-coated quartz crystal microbalance (QCM) sensor for bisphenol A (BPA) detection via surface initiated-ATRP. They first created a two-dimensional inverse opal SiO_2_ layer, containing gold “pinholes”, on a gold coated crystal quartz, by templating a polystyrene colloidal array in a sol–gel matrix and removing the spheres. The resultant honeycomb-like surface (≈610 nm pores) was then functionalized with 2-bromoisobutyryl bromide (initiator). Exposing this transducer to 4-vinylpyridine, BPA, EGDMA, CuBr, and *N*,*N*,*N*′,*N*″,*N*″-pentamethyldiethylenetriamine (PMDETA as ligand) at 48 °C for up to 24 h yielded a conformal MIP coating within the inverse opal network. By exploiting the greatly increased surface area of the inverse opal architecture compared to a flat QCM crystal, the final sensor achieved the sensitive label-free detection of BPA in aqueous samples.

Similarly [121], Xu et al. immobilized 2-bromopropionyl bromide initiator onto magnetic ZnO nanorods (γ-Fe_2_O_3_/ZnO), followed by the controlled co-polymerization of MAA and EGDMA to fabricate fluorescent MIPs for the detection of sulfamethazine. The controlled polymerization yielded uniform MIP layers with a thickness of approximately 200 nm directly on the nanorod surface, exhibiting excellent recognition performance toward the antibiotic.

Surface-initiated RAFT (SI-RAFT) polymerization is a technique in which thiocarbonylthio chain-transfer agents (CTAs) are covalently immobilized on a substrate, and monomers are then polymerized directly from these surface-bound sites. A CTA is a bifunctional molecule—typically a dithioester, trithiocarbonate, or xanthate—that reversibly mediates the transfer of radical activity between growing polymer chains and dormant species, thereby affording good control over molecular weight, polymer architecture, and thickness.

In a representative example [117], Kaur et al. functionalized graphene oxide (GO) sheets with a dithioester RAFT agent that served as surface-bound initiator. Upon addition of methylparathion (MP) as the template, methacrylic acid (MAA) and an appropriate cross-linker were polymerized from these RAFT sites, yielding globular GO@MIP nanostructures. Fourier-transform infrared spectroscopy (FTIR), field-emission scanning electron microscopy (FESEM), and small-angle X-ray scattering (SAXS) confirmed a uniform MIP layer on the RAFT-modified GO. The obtained GO@MIP materials were used to functionalize glassy carbon electrodes to obtain electrochemical sensors for MP. Electrochemical measurements revealed a good sensitivity of the sensor over 0.2–200 ng mL^−1^, with a detection limit of 4.25 ng mL^−1^ and excellent selectivity over structurally related interferents.

Localized polymerization approaches for MIP synthesis, while offering exceptional spatial resolution, are not without limitations (Table 1). The preliminary grafting of initiators, often required, introduces additional synthetic steps that can suffer from low coupling yields and batch-to-batch variability. Furthermore, conventional controlled/living radical protocols are highly sensitive to dissolved oxygen, necessitating rigorous inert-atmosphere techniques that increase experimental complexity and cost. In addition, SI-ATRP requires metal-based catalysts: the inevitable retention of transition metal catalysts within the polymer matrix also poses challenges for downstream applications, particularly those requiring biocompatibility.

### 3.4. Vapor-Phase Synthesis

Most current MIP deposition strategies are based on liquid-phase processes that have been fine-tuned for bulk surfaces. When these methods are applied to nanostructured substrates, significant issues could arise. One of the primary challenges is the limited diffusion of monomers within the nanostructures recesses or pores [122,123], possibly resulting in an uneven growth of the MIP film with non-uniform coatings over depth, particularly on high-aspect-ratio geometries. Due to this, a bottleneck effect can be obtained, hindering the diffusion of other monomer molecules [122,124].

**Figure 20:**
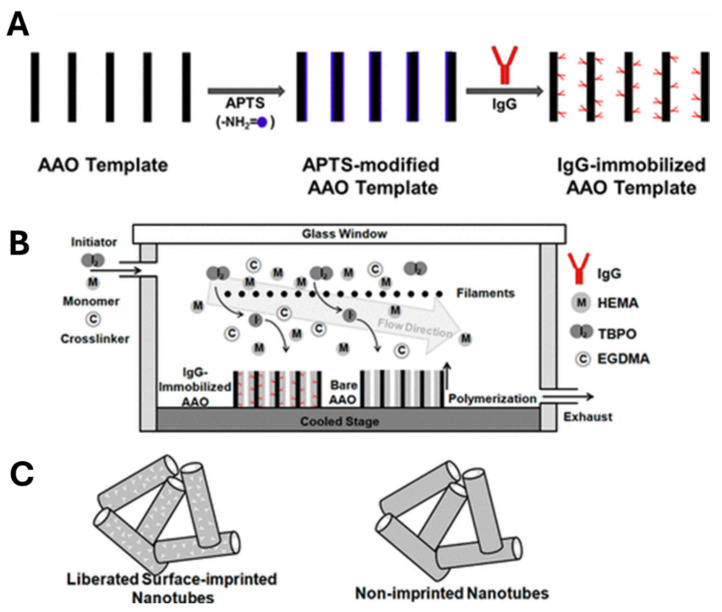
Schematic representation of the fabrication procedure of surface-imprinted and non-imprinted polymeric nanotubes: (**A**) IgG conjugation of AAO membranes, (**B**) polymerization inside IgG-conjugated nanopores and bare AAO by iCVD, and (**C**) liberated surface-imprinted and non-imprinted polymeric nanotubes. Adapted with permission from Ref. [127]. Copyright © 2013, American Chemical Society.

Vapor-phase polymer deposition is a solvent-free approach possibly overcoming such limitations of conventional solvent-based methods [125] and enabling the conformal coating of different (nano)geometrical substrates [126]. An initiated chemical vapor deposition (iCVD) is a specialized vapor-phase polymerization method that employs free radical mechanisms. In a typical iCVD process, monomer and initiator vapors are introduced into a vacuum reactor at pressures in the mTorr range. The initiator molecules are thermally decomposed to generate free radicals by heated filaments (typically 150–300 °C, below the monomer decomposition temperature). These radicals then react with monomers adsorbed onto the substrate, initiating a polymerization reaction that closely resembles its liquid-phase counterpart. Polymerization occurs on the substrate surface, resulting in highly cross-linked, solvent-insoluble polymers. Moreover, the absence of solvents allows for the uniform coating of intricate structures. Due to its simplicity and ability to form polymeric thin films on various substrates, iCVD has been applied in microfluidics, tissue engineering, sensing, photovoltaics, organic electronics, and drug delivery. Nonetheless, its use for MIP synthesis remains relatively limited. In a pioneering study, Ince et al. [127] fabricated MIP nanotubes by combining initiated chemical vapor deposition (iCVD) with a nanomolding strategy (Figure 20). Their approach involved immobilizing IgG molecules within the nanopores of an anodic aluminum oxide (AAO) membrane, followed by the in situ polymerization of poly (2-hydroxyethyl methacrylate) (PHEMA) via CVD inside a custom-built chamber. During deposition, chamber pressure was maintained at 200 mTorr and filament temperature at 280 °C. Monomers, crosslinkers, and the initiator were continuously introduced into the chamber to initiate polymer growth. A polymer layer with a thickness of approximately 80 nm formed along the inner walls of the AAO nanopores. After polymerization, the AAO membranes were immersed in 1 M HCl for 24 h to dissolve the AAO mold and to remove the IgG template from the polymer matrix, resulting in one-dimensional polymeric nanotubes endowed with specific IgG recognition cavities. Notably, the solvent-free nature of this method offers a significant advantage for imprinting solvent-sensitive molecules, although the high deposition temperatures required limit its application to biomolecules.

**Figure 21:**
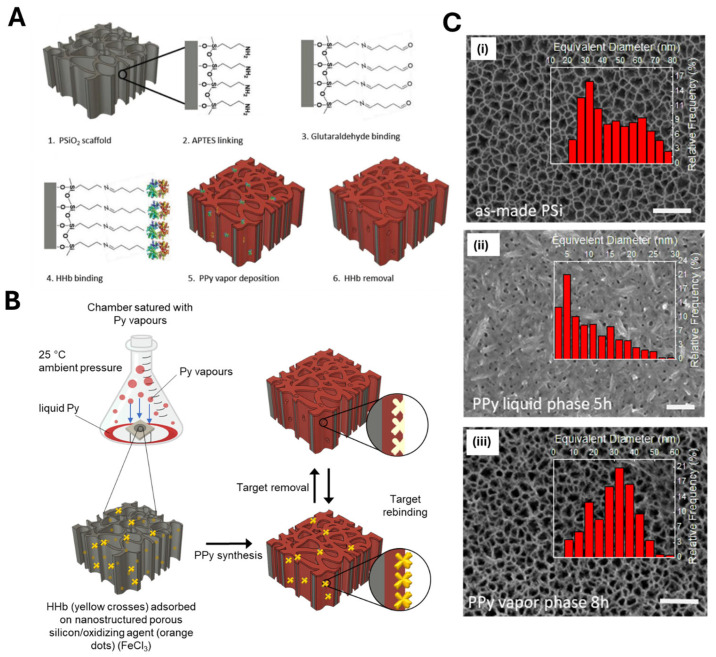
Polypyrrole (PPy) vapor-phase deposition on nanostructured porous silicon oxide (PSiO_2_) scaffolds. (**A**) Sketch of the vapor-phase polymerization process, starting from as-made PSiO_2_ (**1**), functionalized with (**2**) APTES silane, and (**3**) glutaraldehyde, used as suitable linker to anchor the HHb target protein on transducer surface (**4**). Later, the PPy deposition is performed by vapor-phase synthesis. (**B**) Detail of the vapor-phase synthesis approach: after target anchoring, the samples are treated with an oxidizing agent and the exposed Py vapors contained in a closed chamber. Target removal produces the MIP layer. (**C**) SEM images of (**i**) bare PSiO_2_ scaffold, (**ii**) PPy-functionalized scaffolds by liquid-phase polymerization, and (**iii**) PPy-functionalized scaffolds by vapor-phase polymerization. Adapted with permission from Ref. [43]. Copyright © 2023, Wiley-VCH GmbH.

Mazzotta’s group addressed these challenges by developing an innovative room-temperature vapor-phase method to deposit polypyrrole-based MIPs directly onto a nanostructured porous silicon oxide (PSiO_2_) optical transducer [43]. Pyrrole was selected as a functional monomer due to its ability to vaporize at ambient pressure already, while PSiO_2_, used as optical interferometer, was specifically chosen as a challenging substrate to demonstrate the superiority of vapor-phase over conventional liquid-phase polymerization for high-aspect-ratio nanomaterials (>100). First, the target molecule (human hemoglobin, HHb) was covalently anchored to the PSiO_2_ surface (Figure 21). The scaffold was then impregnated with FeCl_3_ and placed in a sealed chamber saturated with pyrrole vapor at ambient temperature. Relying on pyrrole’s high diffusion coefficient in the gas phase (∼10^−2^ cm^2^/s, over 1000× that in solution), rapid and homogenous monomer permeation within the nanoporous structure was achieved, resulting in the deposition of a highly uniform PPy–MIP layer, whose thickness (indirectly estimated as equal to few nanometers) could be modified by adjusting by the polymerization time (from 1 to 8 h).

Interestingly, to demonstrate the method’s versatility, the authors applied the room-temperature vapor-phase approach to the development of an MIP for quercetin (QU) and successfully applied it to the analysis of red and white wines [44]. Additionally, they optimized the process by significantly reducing the polymerization time required for forming the MIP layer within the nanoporous silica matrix—from the initial 8 h to just 30 min—enhancing the overall efficiency and practicality of the technique.

Both sensors were validated in complex matrices—human plasma and serum for HHb and wine for QU—showing good selectivity, with no significant matrix effects or cross-reactivity with interfering components. This performance is largely attributed to the integration of MIPs within the nanostructured transducer. In particular, PSiO_2_ features a honeycomb-like architecture with nanopores averaging 50 nm in diameter. This structure acts as a physical barrier that filters out macromolecules and particulate matter present in complex samples, allowing only smaller analytes to penetrate the pores and interact with the imprinted sites. As a result, MIP selectivity is significantly enhanced.

**Table 1:** Overview of the technique for nanosized MIP synthesis and their direct coupling with nanostructured transducers.

**Technique**	**Advantages**	**Disadvantages**	**References**
nanomolding	precise control over polymer morphology; confined polymerization within nanosized molds yielding to highly ordered nanostructured MIPs; high batch-to-batch reproducibility.	not fully compatible with biological templates (such as proteins, enzymes, etc.) due to the use of organic solvents or high-energy light during the polymerization procedure; not compatible with transducers sensitive to organic solvents; possible mechanical fragility of the obtained polymeric nanostructures; need to optimize the synthetic procedure (such as scaffold removal to avoid damaging the imprinted material).	[41,42,58,69,70,72,73,74,75,76,77]
nanopatterning	customized lithographic techniques for MIP nanostructuring on flat substrates; rapid polymerization; highly conformal 2D and 3D MIP nanostructures with high versatility in MIP shaping.	requires expensive instrumentation (such as cleanroom facilities); complex synthetic procedures with multi-step fabrication processes; limited scaling-up of the procedures.	[78,79,80,81,82,83,84]
electro-polymerization	simple and cost-effective; polymerization in ambient conditions; rapid polymerization; excellent control over polymer features; can be used for MIP deposition on differently shaped transducing surfaces.	limited to conductive substrates; could alter the redox state of electroactive analytes; monomer diffusion, especially in high-aspect-ratio nanostructures, could lead to uneven MIP layer deposition.	[47,48,51,90,92,93,94,95]
dopamine self-polymerization	simple and cost-effective; does not require external catalysts; mild polymerization conditions in aqueous solutions; compatible with biological templates; can be used for MIP deposition on differently shaped transducing surfaces.	polymerization cannot be precisely controlled, affecting reproducibility; highly sensitive to pH changes; aggregation of polymer with nanoparticles formation within solution for longer polymerization times.	[101,102,103]
localized polymerization	exceptional spatial resolution with high control over polymer features (very thin MIP layers achievable); conformal deposition on complex nanostructured transducers; when a controlled/living polymerization is performed, it is possible to stop and restart the polymerization to module the MIP features.	not fully compatible with biological templates (such as proteins, enzymes, etc.) due to the use of organic solvents or high-energy light during the polymerization procedure; not compatible with transducers sensitive to organic solvents; very sensitive to oxygen (polymerization is performed in a controlled environment); a multi-step procedure for MIP synthesis is often required.	[45,61,113,117,120,121]
vapor-phase synthesis	solvent-free synthetic procedures; conformal deposition on nanostructured transducers; high batch-to-batch reproducibility; deposition of very thin and stable MIP layers (a few nanometers); fine control over polymer growth (by modulating the polymerization time).	some of the approaches require vacuum conditions to carry out the polymerization process; when performed under ambient conditions, the use of monomers that are vaporizable at room temperature (e.g., pyrrole) is necessary; the range of monomers that can be vaporized is limited, which restricts material selection.	[43,44,127]

## 4. MIP Applications in Nanosensors

The direct integration of MIP receptors onto transducers yields ready-to-use devices capable of detecting and quantifying specific analytes, even in a complex matrix. Scaling down to the nanosize outstandingly improves the binding kinetics and thus the system’s sensitivity, contributing to its reaching very low detection limits. For these reasons, MIP-based nanosensors are applied in diverse fields. The following section will present selected works regarding their use in environmental protection, food safety, and biological marker detection for diagnostic purposes.

### 4.1. Environmental Monitoring

A deeply concerning environmental contaminant is TNT, persisting in the environment, and having detrimental effects on both the ecosystem and living organisms [128]. Although several analytical techniques are widespread for TNT detection, nanosensors offer a significant advantage regarding sensitivity and selectivity, along with the possibility to perform the analysis directly at the sampling site [129]. In this perspective, Xie et al. [72] imprinted TNT into the walls of silica nanotubes obtained by sol–gel polymerization within alumina membranes used as sacrificial molds. For target detection, they used the change in color produced by the interaction between the target and the MIP nanotubes. The system was characterized by an enhanced uptake by imprinted nanotubes of about 5.6% compared to their non-imprinted counterpart, attributable to the presence of selective and uniform binding sites located at the surface, thus resulting in improved accessibility and binding kinetics. Indeed, when compared to conventional bulk particles, the imprinted nanotubes showed un uptake capacity of 3.6-fold. Moreover, the authors claimed a selectivity coefficient of 5.2-fold for TNT compared to DNT, assessing the extraordinary features of the device.

The massive and often unjustified use of the antibiotic MNZ leads to its easy accumulation in water, with harmful effects on marine populations and contributing to the diffusion of the antibiotic resistance phenomenon [130]. Song et al. [93] functionalized NPAMR by electrodepositing an *o*-PD layer in the presence of MNZ as a template. The removal of the template from the polymer matrix took part in the sensitive electrochemical detection of MNZ, along with the nanoporous NPAMR structure, resulting in a limit of detection of 2.7 × 10^−14^ M. Moreover, the authors proved the sensor’s applicability in detecting the target analyte both in tablets and fish tissues, recording optimal recovery percentages, also corroborated by HPLC analysis on the same samples.

The third-generation fluoroquinolone antibiotic ENR, affecting fish products and persisting in the environment [131], was employed as a target template by Zdunek et al. [74] for the development of a fluorescence sensor based on a surface-imprinted nanofilament MIP. The sensing system relied on the complexation between the target and europium (III) ions on the polymer surface. The presence of ENR binding sites on the surface and the complexation with europium (III) ions were claimed as advantageous over direct ENR detection due to their absorption in the UV region and emission in a wavelength range where interfering effects of the polymer or sample components may occur. Indeed, the sensor detected the target in a concentration range of 0–5 μM, with an LOD of 0.53 μM. Moreover, the system could be reused up to 25 times after a simple regeneration process, validating the feasibility of the proposed strategy.

The common dye MB has found applications not only in the textile industry, but also in the biomedical field, despite being toxic for humans at high concentrations and harmful to the environment; therefore, a detection system to monitor its presence and systems for its removal from wastewater are crucial [132]. In this regard, Wang et al. [133] employed MB as a model target for the development of a SERS sensor based on molybdenum trioxide (MoO_3_) nanorods covered by a polymethacrylic acid layer bearing selective binding sites for the target analyte. The system was able to detect MB below a concentration of 10^−5^ M, with an enhancement factor (EF) of 1.6 × 10^4^, a remarkable value obtained thanks to the cooperation between the MIP and the semiconductive material, where the MIP acted as a gate, contributing to the selective reaching of target molecules to the semiconductor surface.

Recently, our group [45] functionalized nanostructured PSiO_2_ scaffolds, used as optical transducers, with a thin imprinted polymer for propranolol, a β-adrenergic receptor blocker that has become an environmental pollutant due to its extensive use [134,135]. In this case, the preliminary attachment of the photo-iniferter CDTPA was performed, which drove the MAA surface-initiated polymerization under green light. After the target’s removal, binding sites for propranolol were imprinted in the polymer matrix, and the sensor linearly responded to the target rebinding in a concentration range of 5–100 μM, resulting in a limit of detection of 1.4 μM, as well as satisfactory selectivity, reproducibility, and reusability. More importantly, the sensor was able to detect propranolol in different media, such as acetonitrile, ultra-pure water, and tap water, with comparable results upon a simple pretreatment of the sample.

### 4.2. Food Monitoring

Food containers, bottles, and tools for microwave cooking are among the materials whose manufacturing is based on BPA, a synthetic chemical compound whose exposure has been correlated to several human organ dysfunctions, such as endocrine and reproductive system disruption [136]. Griffete et al. [70] developed a BPA stimuli-sensitive optical sensor based on an MIP-based hydrogel inverse opal, to which a planar defect layer was introduced to improve sensitivity. Interestingly, the BPA molecules rebinding to the imprinted polymer produced a stimulus to which the system mechanically responded, resulting in a Bragg peak red shift. This architecture resulted in a sensitivity of 70 nm μM^−1^, which was greater than that of the defect-free structure. Additionally, a figure of merit (FOM) of 2.8 μM^−1^ was claimed, based on the ratio between the linear regression slope and the FWHM and resulting from the combination of higher Bragg peak shifts and lower FWHM. Overall, the presence of a defect layer proved the construction of a sensitive, feasible, and selective architecture for the label-free detection of BPA, opening new and versatile ways to functionalize inverse opal hydrogels.

Triazophos (TAP), a common pesticide for aquatic and land management, has shown its efficacy in several types of crops, although it is particularly harmful to fish and honeybees. Li et al. [137] functionalized a GCE with CNTs decorated with AuNPs, around which an electro-polymerized *o*-hydroxyphenol polymer (PHP) was deposited and non-covalently imprinted with TAP. Electroactive and electrocatalytic CNTs, 50 nm in diameter, were preliminarily functionalized with carboxylic groups, which interacted with the hydroxyl group of the functional monomer, thus acting as a boost for the monomer’s electro-polymerization. On the other hand, AuNPs improved the system conductivity and surface area, resulting in the electrode’s enrichment with the target analyte. The as-made sensor detected TAP in a wide concentration range (10^−7^–10^−5^ M), recording a low LOD (9.3 × 10^−8^ M). Binding site selectivity was confirmed against different pesticides, and TAP was detected even in spiked vegetable specimens with high recovery percentages.

Food additives, despite being employed to improve the features of processed animal-derived food, recently emerged as endocrine-disrupting chemicals (EDCs) due to their ability to interfere with endocrine signaling even at the ng/L scale. Among them, 17β-estradiol (E2) possesses the most substantial estrogenic-like effect, with a reported negative impact on the prostate in men and breast tissue in women, as well as infertility and obesity [138,139]. Mugo and Lu [140] grafted an E2-imprinted polymethacrylate polymer over a nanoporous biogenic silica (BS) surface derived from phytoplankton. BS was characterized by a rough surface, providing high surface areas and 200 nm diameter pores, thus resulting in a particularly suitable material for SERS detection. The MIP layer over BS possessed a 50 μm thickness and high porosity, allowing rapid mass transfer kinetics for the target’s interaction with binding sites derived from the imprinting process. The sensor was able to detect E2 (0.1–4 ng/mL) with an LOD of 0.073 ng/mL. Moreover, the nanosensor proved selective against E2 structurally similar compounds, such as estrone and estriol, and suitable for the target analyte detection in milk samples.

Fatty acid esters of 3-chloropropane-1,2-diol (3-MCPD), improving soy sauce-based products’ features, are currently recognized as possibly carcinogenic to humans, while adverse effects on rat liver, kidney, and reproductive system were reported [141]. Cheng et al. [91] synthesized poly (*p*-aminothiophenol) (*p*-ATP) imprinted with 3-MCPD on a GCE previously coated with NPG, characterized by a uniform rough structure and homogenously distributed cavities, which improved the electrocatalytic effect. The sensor selectively detected the target analyte in a wide concentration range (10^−16^–10^−7^ M) by differential pulse voltammetry (DPV), with an LOD of 3.5 × 10^−17^ M. The applicability of the sensor was also tested in soy sauce samples, resulting in high recovery percentages.

The flavonoid QU, possessing antioxidant and anticancer effects, plays a crucial role in wine’s sensory attributes, contributing to its astringency, bitterness, and overall flavor profile. However, high concentrations of quercetin can lead to sediment formation over time, potentially compromising the clarity and commercial viability of wine [142,143]. Our group [44] recently developed a QU sensor based on an imprinted PPy matrix obtained by vapor-phase in the inner structure of nPSi. The sensor was tested for QU detection in water and water/ethanol mix, with a dual linear response (LOD = 0.7 μM) due to different binding site populations. Red and white local wine samples were tested for sensor applicability, and the results were corroborated by HPLC analysis.

### 4.3. Biomarker Detection

The current trend in cardiac muscle injury diagnosis relies on measuring several tissue-specific markers. The protein MYO, characterized by the presence of a heme prosthetic group, elevates its concentration above the physiological range (17.4–105.7 ng/mL) in myocardial necrosis and renal insufficiency and serves as an early sensitive biomarker [144]. Linares et al. [58] carried out a nanomolding procedure on a porous alumina structure where the template was preliminarily immobilized, followed by the UV-mediated synthesis of rodlike-structured imprinted nanofilaments by employing HEMA or methyl methacrylate (MMA) as functional monomers. The nanofilaments were shown to be stable and very adhesive, and HEMA polymerization resulted in a hydrophilic imprinted polymer with an imprinting factor equal to 7. The same group [78] later combined microscope projection photolithography with nanomolding and molecular imprinting to develop nanofilament dot arrays for the same target analyte for the direct fluorescence detection after its immobilization in the nanoporous alumina substrates, resulting in an imprinting factor of 4.3 and a selective system for MYO binding. The authors stated the protocol as rapid and economically affordable for developing sensitive and selective detection systems.

Human hemoglobin (HHb), a red blood cell globular protein that transports molecular oxygen, is a crucial biomarker for the evaluation and management of different diseases as anemia, a clinical condition characterized by a reduction in HHb concentration or red blood cell number [145,146]. Our group [43] recently synthesized a HHb-imprinted polypyrrole PPy on a nanostructured PSiO_2_ scaffold, serving as an interferometric transducer. The strategy relied on the PPy vapor-phase deposition on the substrate as an alternative to liquid-phase polymerization, which often results in a non-homogeneous coverage of the platform, achieved by placing the substrate in a pyrrole vapor-saturated chamber for 8 h, forming a thick layer of the polymer, visible to the naked eye. The subsequent template removal gave rise to two distinct populations of binding sites, such that the sensor responded distinctly, with a higher-sensitivity region and a lower-sensitivity region. However, the overall LOD was 0.024 mg/mL. At the same time, the imprinting factor, as a comparison between imprinted and non-imprinted polymer, was calculated as 13.1, which could undoubtedly be considered an extraordinary value considering the imprinting of a protein. Finally, the sensor proved selective against common interfering proteins and applicable in HHb detection in spiked human plasma and artificial serum.

## 5. Conclusions

The recent progress in the field of nanosensors, promoting their widespread use in several application fields, including precision medicine and wearable devices, makes the development of techniques enabling reliable receptor/transducer integration urgent. This aspect needs to be critically evaluated when such integration involves the use of molecularly imprinted polymers as artificial receptors, which nowadays represent a valid alternative to their biological counterparts, undergoing a revolutionary transformation due to their high potential in terms of selectivity, robustness, stability, cost, and flexibility.

This review aims to present an overview of the technologies recently developed for achieving the direct and reliable integration of MIPs with transducer at the nanoscale, specifically focusing on strategies enabling MIP nanomanufacturing on bulk transducers (namely, MIP nanopatterning and nanomolding techniques) and on strategies enabling the highly conformal deposition of MIPs as ultrathin layers on nanostructured transducer surfaces (namely, localized polymerization techniques, electro-polymerization, and vapor-phase polymerization). In both cases, the resulting nanosensors present enhanced performance deriving from nanoscale-related processes, such as high surface area, more accessible binding sites, faster binding kinetics, and low limits of detection. A discussion of the benefits and possible limitations of each presented approach is provided, and successful examples of their application in the detection of environmental pollutants, food contaminants, and clinical biomarkers are also given.

Although high accuracy and excellent spatial resolution can be achieved by most MIP nanostructuring techniques, effectively enabling efficient and stable MIP–transducer integration, some additional efforts are still required to realize MIP-based nanosensors’ transformative potential.

The fabrication of MIP–transducer integrated systems at the nanoscale demands extremely high precision; however, this level of complexity and accuracy also constrains the overall device size and the production speed. The automation and integration of the entire process, from nanostructure preparation to device assembly, would be greatly beneficial to reduce costs and shorten development timelines, thus paving the way for faster real-world translation and large-scale commercialization.

Advancements are needed to improve process compatibility with flexible materials to achieve further applications of MIP-based nanosensors in flexible/wearable devices. Ideally, the development of new nanomanufacturing techniques and materials should occur in a coordinated manner through interdisciplinary collaboration, including materials science, nanofabrication techniques, bioengineering, electrical engineering, and artificial intelligence.

One of the major challenges in applying MIPs to real-world samples is their potential cross-reactivity with matrix constituents, particularly when interfering species are present at concentrations several orders of magnitude higher than the target analyte. This limitation can be effectively mitigated by integrating MIPs with nanostructured transducers. In particular, nanoporous architectures—such as those found in photonic crystals—act as selective physical barriers that exclude large macromolecules and particulate matter while allowing smaller analytes to reach the imprinted recognition sites. This structural selectivity significantly enhances the overall performance and specificity of MIP-based sensors in complex matrices.

Notably, this advantageous feature is also shared by other nanoporous transducers, such as nanoporous gold (NPG) and inverse opal structures obtained via nanomolding techniques.

In conclusion, the integration of nanosized MIPs with nanostructured transducers represents a powerful strategy to overcome the traditional limitations of MIP-based sensing, particularly in complex analytical environments. Future efforts should aim at further optimizing these hybrid systems to achieve improved reproducibility, scalability, and applicability in real-time and point-of-care diagnostics.

## Data Availability

Not applicable.
